# Controlling endemic foot-and-mouth disease: Vaccination is more important than movement bans. A simulation study in the Republic of Turkey

**DOI:** 10.1016/j.idm.2025.02.006

**Published:** 2025-02-13

**Authors:** Glen Guyver-Fletcher, Erin E. Gorsich, Chris Jewell, Michael J. Tildesley

**Affiliations:** a Zeeman Institute for Systems Biology and Infectious Disease Epidemiology, Research, University of Warwick, Coventry, CV4 7AL, UK; b School of Life Sciences, University of Warwick, Coventry, UK; c Mathematics Institute, University of Warwick, Coventry, UK; d Department of Mathematics and Statistics, Lancaster University, Lancaster, UK

**Keywords:** Cattle, Foot-and-mouth disease, Disease control, Endemic

## Summary

In this article we present a spatially-explicit stochastic metapopulation model to simulate the spread and control of foot-and-mouth disease (FMD) in an endemic setting. We parameterise and validate the model using detailed outbreak data from the Republic of Turkey, 2001–2012. Subsequently, we assess the efficacy of ring vaccination, mass vaccination, and livestock movement restrictions with regards to incidence-reduction and likelihood of eradication.

Key findings show that ring vaccination and mass vaccination are most effective for controlling endemic FMD; livestock movement controls do not lead to eradication on average. Combined biannual mass vaccination and 10 km ring vaccination around detected farms emerged as the optimal approach to maximise the probability of FMD elimination within a few years. The probability of disease detection, radius of ring vaccination, and coverage of mass vaccination are most critical to optimize for this policy. Our results suggest countries wishing to control the disease within their borders should focus on comprehensive surveillance and vaccination campaigns as their main policy goals. In summary, vaccination-based policies are more effective than movement restrictions in the endemic context.

## 1 Introduction

Foot-and-Mouth Disease (FMD) is an acute disease of cloven-hoofed livestock, characterised by vesicular lesions on the feet, tongue, snout and teats, as well as fever and lameness. The Foot-and-Mouth Disease Virus (FMDV), of the family Picornaviridae and genus *Apthovirus*, is highly contagious and infects many domesticated livestock species, although concern concentrates on cattle and pigs [Arzt et al., 2011]. Although the disease currently circulates predominantly in Asia and Sub-Saharan Africa, there remains a threat of reintroduction to the livestock industries of FMD-free countries. FMD, therefore, imposes direct and indirect costs on both free and non-free regions [Knight-Jones & Rushton, 2013].

Many regions where FMD is regionally-persistent (i.e. endemic regions) even if not always locally wish to control the disease within their borders or within sub-regions according to the FMD Progressive Control Pathway (PCP) [Sumption et al., 2012]. Epidemiological modelling can provide insights into the dynamics of the disease within a region, and allows the attempted optimisation of control policies with limited expenditure of scarce resources. However, few spatially explicit and mechanistic modelling efforts have looked at endemic regions, despite the vast majority of FMD cases taking place in these settings [Zaheer et al., 2020; Ringa & Bauch, 2014; Kim et al., 2016; Schnell, 2019; Guyver-Fletcher et al., 2022]. The complexity of populations where pre-existing immunity is widespread (but extent is unknown) and multiple serotypes are circulating - combined with a common lack of data in endemic regions - means modelling endemic FMD remains a challenge.

The Republic of Turkey is a country where FMD remains endemic [FAO, 2023], but high-quality data on disease circulation is available. Efforts to control the disease are long-standing - although exact numbers are not available, Ozturk et al. (2020) quantified that animal-level prevalence has been reduced from 45% in 2008 to 5% from in 2018. Turkey offers an opportunity to develop models of endemic FMD, probe the dynamics of the endemic state, and assess control policies, using mathematical epidemiological models.

This study aims to: (1) develop and parametrise a model of endemic FMD with the data available; (2) use said model to assess how likely the current controls are to lead to eradication of FMD; and (3), probe which aspects of those control policies are most important to controlling of the disease. We construct, test and utilise a metapopulation compartmental mathematical model of the disease, fit it to available data, and use it to simulate both the spread of the disease and various control policies.

## 2 Materials and methods

### 2.1 Data

The data available from the Republic of Turkey consisted of: farm outbreak incidence data (covering 2001–2012); cattle shipment data (covering 2007–2012); farm location data; and farm cattle headcount data (2010 census). A description of these data has been published previously in [Dawson, 2016].

**Fig. 1:**
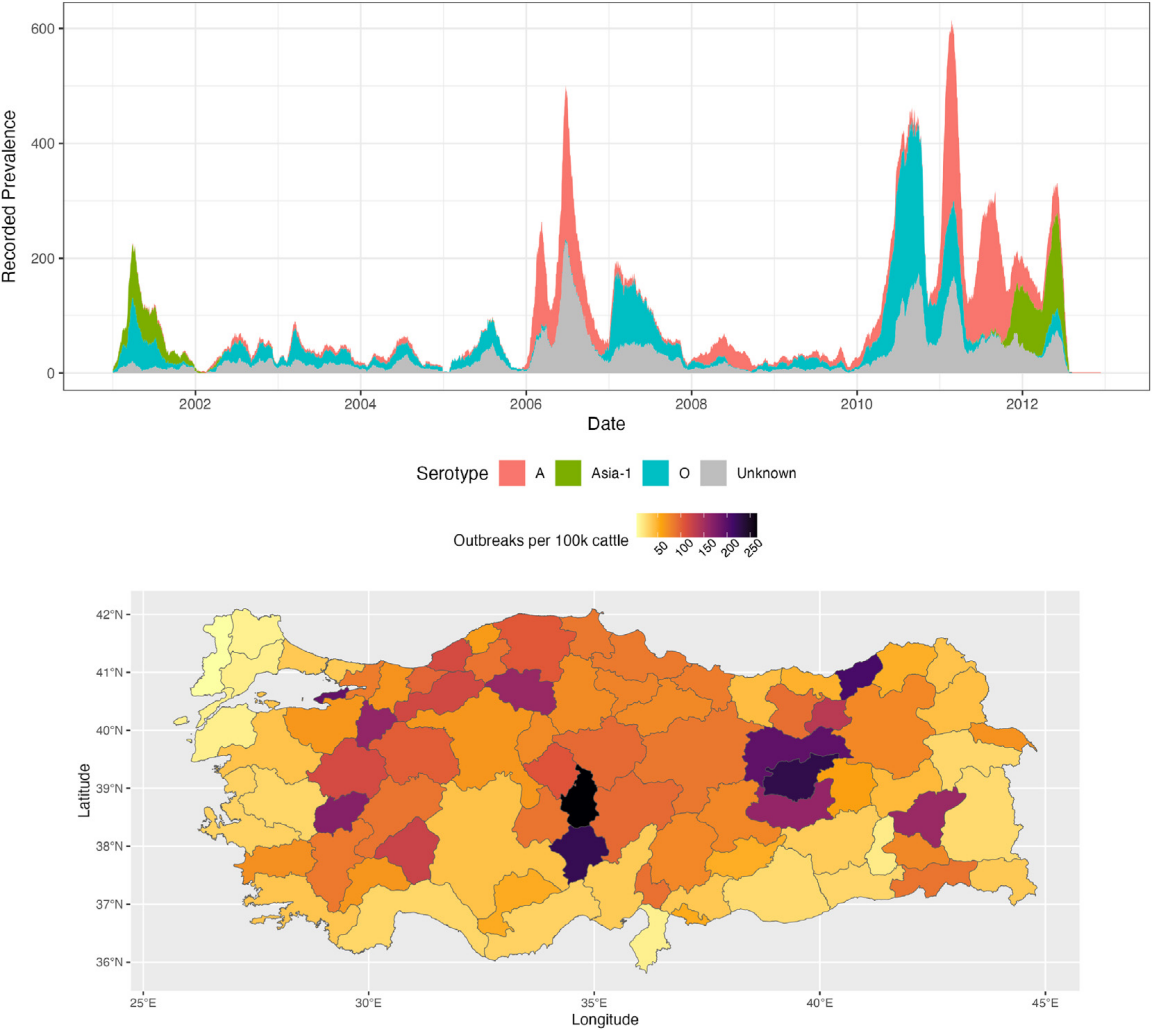
**Time and spatial distributions of FMD in Turkey, 2001**–**2012.** The top figure shows the absolute number of recorded ongoing FMD outbreaks by serotype over this period, estimated by taking the recorded start date and end of quarantine date for each outbreak. There are clear epidemics in 2001, 2006, and 2010–12. The bottom figure shows the spatial distribution of these outbreaks, normalised by number of cattle, with each province highlighted according to how many outbreaks per 100k cattle were in each province. Erzurum Province, which we choose to model, is in orange; although it has by far the greatest number of outbreaks, it also has a relatively great population of cattle. Many central provinces are intermediate in outbreaks per 100k cattle, and European Thrace is very low.

Fig. 1 shows a summary of the temporal and spatial distributions of the recorded farm-level FMD outbreaks. We see three separate serotypes (O, A, Asia-1), and a proportion of un-typed outbreaks. There are clear epidemics in 2001, 2006/7, and a more durable increase in incidence from 2010 onwards. The dominant serotype differs for these peaks; serotype A dominates 2006/7 while serotype O dominates 2010, and there is a resurgence of Asia-1 in 2012. In total 9282 infected farms were recorded over this period, 2879 being of serotype O, 2651 of serotype A, 905 of serotype Asia-1, and 2847 for which the serotype was unidentified.

There is also clear spatial heterogeneity in recorded outbreaks, which tend to cluster in select provinces. Fig. 1 shows the normalised number of outbreaks per 100k cattle of each province. In the east, Erzurum province has many recorded outbreaks, albeit with a greater number of cattle, but there are some provinces with more outbreaks than might be expected taking into account their cattle population (e.g. Nevsehir, Yalova, Tunceli provinces). Though not displayed visually, there is a central band of high-incidence provinces including Ankara, Afyon, Kayseri, Silvas, Kastamonu and Samsun. European Thrace in the west exhibits very low incidence over this decade, however, and is currently acknowledged as free of the disease. This hetereogeneity in incidence is correlated with variation in the cattle population in our data. Two of the highest incidence provinces, Erzurum and Ankara, are also two of the provinces with the highest cattle population., and the state-level correlation of the sum of recorded outbreaks and cattle population of that state is 0.728.

**Fig. 2:**
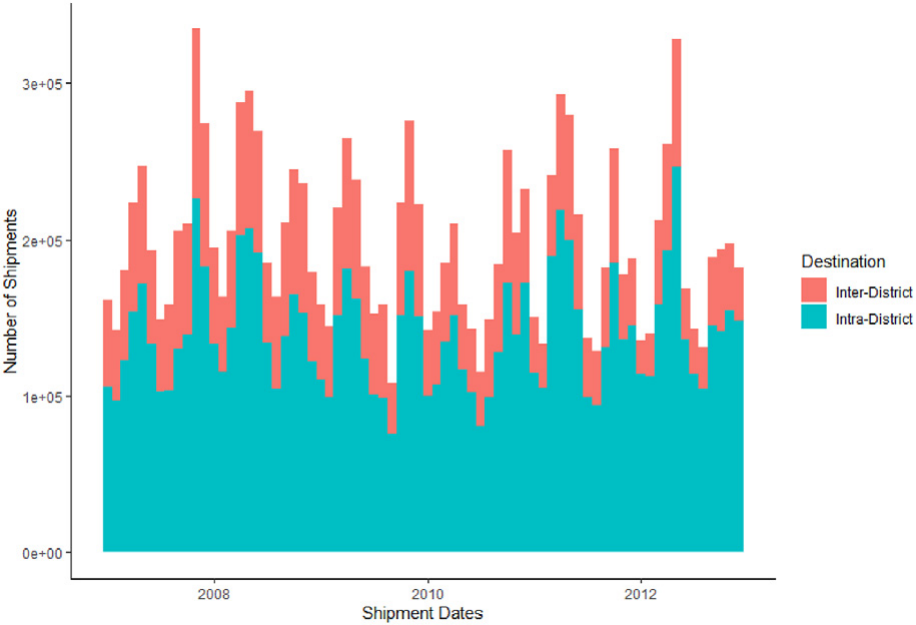
Monthly Cattle Shipments in the Republic of Turkey over the period 2007 to 2012, when data are available. Blue indicates the number of within district shipments, and red the number between districts. There is a clear seasonal pattern of shipments peaking every year for the festival Kurban Bayrami. Within-district shipments make up the clear majority of all cattle shipments recorded.

Cattle shipment records were also available: these recorded the number of cattle shipped between a source and destination farm, and on which day this occurred. There were 14,261,447 records of this type, spanning the entirety of Turkey and covering the years 2007–2012. Fig. 2 demonstrates the high number of cattle shipments occurring every month, as well as the clear seasonal variation and the pattern due to the religious festival of Eid-al-Adha. This festival involves animal sacrifice, consequently there is an increase in animal movements. Incorporating these records allowed for the seasonality of such shipments to be explicitly modelled. The median and mean shipment distance for the entirety of Turkey was 28.3 km and 94.7 km, due to the long tail of very long distance shipments.

The data also make clear the local nature of most cattle movements – the majority of the recorded cattle shipments were between farms in the same district, with only a minority going further. The vast majority of these shipments moved only one animal, with a median of 1 and a 99th-percentile shipment size of 30 animals.

These data were made available as separate data-sets, requiring cross-referencing with each other. After cross-referencing and cleaning, we obtained 40,208 farms with an associated cattle headcount and point location. As computational complexity in our framework scales exponentially with the number of simulated farms, we simulate a single province (Erzurum province) with 1108 farms, 605,177 cattle, and 253 outbreaks between 2007 and 2012. This provides an average of 0.228 outbreaks per farm over the 5 year period.

### 2.2 Model structure

**Fig. 3:**
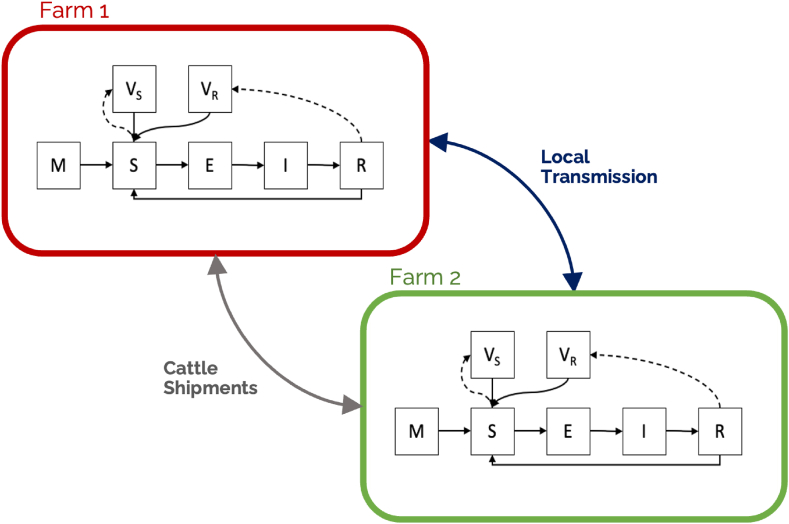
**Model Schematic**. Each farm is treated as an epiunit, and animals within are classified into one of the disease-relevant classes and can proceed through them along the arrows. S, E, I and R, correspond to the basic Susceptible, Exposed, Infectious, and Recovered classes. M indicates Maternally Immune; *V*_*S*_ indicates Vaccinated from Susceptible (i.e. no prior immunity), and *V*_*R*_ indicates Vaccinated from Recovered (i.e. prior immunity). It is assumed that M, E and I can not be successfully vaccinated. Transition rates between classes are described in Eq. (1), with parameters in Table 1. Between-farm transmission is carried out through a dispersal kernel *K*(*d*_*ij*_) (Eq. (2)) and through animal shipments between farms. Note that for simplicity, arrows for natural birth rates, death rates and sub-classes are not included here.

We utilise a stochastic spatial metapopulation model, where each farm is considered a separate population and the within-farm and between-farm dynamics are modelled interdependently. Fig. 3 gives a schematic overview of this. On each farm, we divide the cattle into a number of mutually-exclusive epidemiological classes, consisting of susceptible (*S*), exposed (*E*, i.e. infected but not yet infectious), infectious (*I*), and removed (*R*). In addition, we have a class *M* representing maternally immune individuals, and split the vaccinated class into Vaccinated-Susceptible (*V*_*S*_) and Vaccinated-Recovered (*V*_*R*_) to better capture the dynamics of vaccinating previously-exposed animals. We define the number of cattle in class *q* on farm *i* at time *t* as $X_{it}^{q}$, with the farm size given by $N_{it}=\sum_{q}X_{it}^{q}$ for *q* ⊂ {*M*, *S*, *E*, *I*, *R*, *V*_*S*_, *V*_*R*_}.

#### 2.2.1 Within-farm model

At the beginning of the model timeline, it is assumed that all animals within each farm are susceptible, but then progress between states *q* and *r* according to rate $\lambda_{i}^{qr}\left(t\right)$, so that

(1) $$\begin{array}{ll} \lambda_{i}^{MS}\left(t\right) & =\mu X_{it}^{M}\\ \lambda_{i}^{SE}\left(t\right) & =\beta X_{it}^{S}X_{it}^{I}/N_{it}\\ \lambda_{i}^{EI}\left(t\right) & =\sigma X_{it}^{E}\\ \lambda_{i}^{IR}\left(t\right) & =\gamma X_{it}^{I}\\ \lambda_{i}^{RS}\left(t\right) & =\phi_{R}X_{it}^{R}\\ \lambda_{i}^{V_{S}S}\left(t\right) & =\phi_{V_{S}}X_{Si}^{V}\left(t\right)\\ \lambda_{i}^{V_{R}S}\left(t\right) & =\phi_{V_{R}}X_{Ri}^{V}\left(t\right). \end{array}$$

where $\mu ,\beta ,\sigma ,\gamma ,\phi_{R},\phi_{V_{S}},\phi_{V_{R}}$ are parameters described in Table 1.

**Table 1:** Relevant parameters of the model, with value(s).

Parameter	Description	Value(s)	Source(s)
*α*	Birth rate	2%/year	
*β*	Transmission	6/11 transmissions per contact	[Orsel et al., 2005; Orsel et al., 2007; Tadesse et al., 2019]
*γ*^−1^	Mean infectious period	11 days	[Yadav et al., 2019]
$\phi_{R}^{-1}$	Mean time recovered	250 days	[El-Sayed et al., 2012; Doel, 2005]
*μ*^−1^	Mean duration of maternal immunity	120 days	[Nicholls et al., 1984]
*σ*^−1^	Mean latent period	1.5 days	[Yadav et al., 2019]
Ω_*d*_	Infection mortality rate	2%	[Şentürk & Yalçin, 2008]
Ω_*n*_	Natural mortality rate	2%/year	
$\phi_{V_{S}}^{-1}$	Mean time vaccine-immune (susceptible)	150 days	[Doel, 2003; Cox et al., 2010; Knight-Jones et al., 2015]
$\phi_{V_{R}}^{-1}$	Mean time vaccine-immune (recovered)	max$\left(\phi_{R},\phi_{V_{R}}\right)$	[Doel, 2003; Cox et al., 2010; Knight-Jones et al., 2015]
*ρ*	vaccine delay days	4 days	[Barnett & Carabin, 2002]

*ξ*	Inter-farm per-capita transmission	6.806e-6	[Keeling et al., 2001; Tildesley et al., 2006]
*ν*	Kernel Scale parameter	1	[Jewell et al., 2009]
*η*	Kernel shape parameter	2	[Jewell et al., 2009]

Additionally, a natural death rate Ω_*n*_ is taken from all classes, and a disease-associated death rate Ω_*d*_ is taken from class *I*. In endemic situations with high FMD seropositivity, newly born calves may either receive maternally-derived antibodies (hence protection from infection and vaccination) or not (susceptible). To represent this, births occur at a rate *αN*_*it*_ and are randomly assigned class *M* with probability $\left(X_{it}^{R}+X_{it}^{V_{S}}+X_{it}^{V_{R}}\right)/N_{it}$ (i.e. the proportion of the farm population that is seropositive) and class *S* otherwise.

Stochastic simulation of disease progression was done using the *τ*-leap approximation, which draws the number of events (such as infection, recovery, etc.) in a given time-step *δt* from a Poisson distribution [Gillespie, 2001]. A time-step (*δt*) of 1 day was used to allow synchronisation with other data such as livestock shipments. To provide non-exponential distributions of class sojourns, several classes have multiple sub-classes [Keeling & Rohani, 2008]. The *M* class has 3, the *R* and both *V*_⋅_ classes have 20; this also has the useful effect of ensuring animals cannot immediately begin leaving a class they've just entered, effectively enforcing a minimum length of time (3*δt* and 20*δt* respectively) animals are expected to reside in these classes. Due to a lack of age data, there is no age-stratification of the classes.

#### 2.2.2 Between-farm transmission

The virus is transmitted between farms either by local spread or the shipment of cattle. The probability of infection by local spread *p*_*i*_(*t*) on farm *i* from all other farms *j* (where $X_{jt}^{I}>0$) is defined as

(2) $$\begin{array}{ll} p_{i}\left(t\right) & =1-e^{-\xi \underset{j\neq i}{\sum }X_{jt}^{I}K\left(d_{ij}\right)},\\ K\left(d_{ij}\right) & =\frac{1}{1+{\left(\frac{d_{ij}}{\nu }\right)}^{\eta }}. \end{array}$$

where *d*_*ij*_ is Euclidean distance between farm centroids, *ξ* is a baseline farm-farm transmission rate, and *ν*, and *η* govern how local spread decays with distance between farms. We assume *ξ*, *ν*, and *η* are unknown and are estimated as described below.

For each recipient farm *i*, we draw the number of cattle infected by local spread per day, $Z_{it}^{SE}$, from a Binomial random variable, such that $Z_{it}^{SE}∼\text{Binomial}\left(X_{it}^{S},p_{i}\left(t\right)\right)$.

Initial *ν* and *η* parameters were chosen to match the dispersal kernel from [Jewell et al., 2009]; this kernel has been used to flexibly describe the spread of FMD in many different regions such as the UK and Japan [Probert et al., 2018; Jewell et al., 2009]. It has also been used in the USDOS model of disease spread in the USA, although the lack of FMD there prevents its validation [Tsao et al., 2020]. For computational speed we use the grid algorithm of [Sellman et al., 2018].

Livestock shipments are simulated on a daily basis and replay the available animal movement records. Doing this allows the seasonality of those shipments to be accurately captured, but limits the period which can be modelled to 2007–2012. Shipments are modelled as moving a random sample without replacement from the animals at the source farm to the target farm, hence the probability of selecting at least one infected animal is proportional to the shipment size and number of infected animals in the source farm.

Vaccination (either *S* → *V*_*S*_ or *R* → *V*_*R*_) occurs in the model only in response to a control policy option, either ring vaccination or mass vaccination. Farms are identified as requiring vaccination with probability equal to the input vaccine coverage and for ring vaccination an additional requirement of being within a specified radius of a known infected farm. Identified farms are added to a queue and then vaccination proceeds until the daily vaccination capacity is reached. Ring vaccination follows an ’outside-in’ strategy; there is no ordering for mass vaccination. Vaccination of a farm leads to both susceptible and recovered individuals proceeding to their respective vaccinated classes, in proportion to the efficacy of the vaccine and after a fixed delay of *ρ* days. Maternally immune (M) animals are assumed not to be vaccinated, and currently infected animals (classes E and I) are added to the count of vaccines used but do not proceed to the vaccinated class due to their current active infection. The realised vaccine efficacy for each farm *VE*_*i*_ is drawn from a normal distribution characterised by the provided mean and standard deviation of the vaccine efficacy. class *V*_*S*_ wanes at rate $\phi_{V_{S}}$, and class *V*_*R*_ at max(*ϕ*_*R*_, $\phi_{V_{R}}$). It should be noted that this model assumes ’all-or-nothing’ vaccination, where the vaccine either provides full protection or none at all - this tends to underestimate required herd-immunity thresholds for eradication as in reality vaccines provide imperfect protection (i.e. ’leaky’ vaccines).

The simulated control policies are mass vaccination (M), ring vaccination (R) and livestock shipment controls (S). Both ring vaccination and shipment controls occur in response to a detected infection and occur within a radius of the infected farm. Livestock shipment controls prevent the movement of cattle to or from farms within a radius of a detected infected farm, with probability equal to the input coverage. Mass vaccination occurs on a defined interval, and pulse vaccinates all of the farms in the region whenever that interval has been reached.

Simulation and analysis code is available at https://zenodo.org/records/11192752.

### 2.3 Model fitting

Model fitting was done using the Approximate Bayesian Computation Sequential Monte Carlo (ABC-SMC) algorithm as described in [Toni et al., 2009], and later described in [Minter & Retkute, 2019]. The initial tolerance was set to *∞*, and the tolerance for following generations calculated using the bisection method outlined in the supplementary material of [McKinley et al., 2018], with minimum, maximum and mean quantiles of 45%, 55% and 50%. The distance metric was the sum of the squares *SS* (Eq. (4)) of the simulated incidence against the “true” or observed incidence, with incidence taken as the count of newly recorded infected farms on each day.

Let $\chi \left(i,t\right)=X_{it}^{E}+X_{it}^{I}$, indicating whether farm *i* is infected at time *t*. We then define an indicator function I(i,t) for incidence, which returns 1 when farm *i* is newly infected at time *t*, and 0 otherwise.

(3) $$I\left(i,t\right)=\left\{\begin{array}{ll} 1\; & if\chi \left(i,t-1\right)=0,\chi \left(i,t\right)>0\\ 0\; \end{array}\right)$$

Note that this definition means that if a farm is infected multiple times in a short period (for example if the first introduction dies out) it will be counted as multiple cases. A more robust statistic based on number of animals infected could be considered, but we do not have reliable empirical data on this to compare to.

We then use this to define our summary statistic, *SS*:

(4) $$SS=\overset{t_{\max}}{\underset{t=1}{\sum }}{\left(\left(\overset{F}{\underset{i=1}{\sum }}\overset{t}{\underset{s=1}{\sum }}I\left(i,s\right)\right)-\left(\overset{F}{\underset{i=1}{\sum }}\overset{t}{\underset{s=1}{\sum }}y_{is}\right)\right)}^{2}$$

where *F* is the number of farms, and *y*_*is*_ = 1 if a new outbreak was observed on farm *i* on day *s* and 0 otherwise. Our summary statistic is the sum of the squared differences between the observed and simulated cumulative incidence.

#### 2.3.1 Identifiability analysis

Identifiability analysis was performed to ascertain which parameters were recoverable from the data using ABC-SMC. Parameter values were drawn 5 separate times from a joint prior distribution, and these values used to generate synthetic outbreaks. We then ran the ABC-SMC algorithm on the synthetic data, attempting to recover the ’true’ parameter values from our priors. We considered a parameter as recovered if the algorithm converged on the ’true’ parameter values. The details of this analysis can be found in the supplementary material.

#### 2.3.2 Parameter estimation

Parameter estimation was carried out using ABC-SMC to estimate the parameters *ξ*, *ν* and *η* (Eq. (2)) of the local-spread kernel, as the identifiability analysis did not allow identifying *β* or *ϕ*_*R*_. As before, 500 particles were used per generation and the prior distributions described in Table 2. The sum of squares *SS* was used as a summary statistic.

**Table 2:** **Prior Distributions for parameter identifiability and inference**. The prior distributions used to generate ”true” values for parameter identifiability analysis and for parameter identifiability.

Parameter	Parameter Description	Distribution
*β*	Acutely infectious transmission	*U*(0.135, 1.8)
*ϕ*_*R*_	Average duration of recovered state	*U*(150, 550)
*ξ*	Inter-farm per capita transmission	*N*(6.8*e* − 6, 6.8*e* − 6)
*ν*	Kernel scale parameter	*N*(1.0, 0.75)
*η*	Kernel shape parameter	*N*(2.0, 0.75)

For each ABC-SMC particle the model was simulated once and compared to the outbreak incidence data for Erzurum Province over the 5-year period 2007–2012. A ”burn-in” period of 365 days was simulated for each particle to allow endemic circulation and population immunity to occur, before the result was compared to the endemic state observed in Erzurum. This burn-in period data was discarded and not used for comparison or analysis.

As there were control policies for FMD in place during this period, the fit attempt also needed to include control policies and surveillance to avoid underestimating disease transmission. These were parameterised using estimates from Turkish collaborators. During this period surveillance for FMD in Turkey was almost entirely passive, relying on farmer reporting. Vaccine efficacy was drawn from a normal distribution with mean 0.65 and standard deviation of 0.05 (i.e. VE 65%, s.d 5%), as the vaccine in use at the time was known to be approximately this effective [Knight-Jones et al., 2014]. Mass vaccination was also in place during this period, occurring every 6 months (182 days), as were reactive ring vaccination and reactive shipment controls within 10 km of a known infected farm. These were assumed to occur in a 10 km radius with 90% coverage and for the shipment controls to last 7 days. Detection was assumed to identify 95% of infected farms. A fixed delay period of 3 days between infection and detection was assumed, which was the average delay in the assumed start dates to confirmation dates for those outbreaks where this was provided. However, there are no data for vaccination or reporting coverage.

### 2.4 Controls

The described model and numerical parameter estimates were then used to investigate the efficacy of different control strategies in the Republic of Turkey from a situation of endemic FMD. To generate endemic FMD, 2500 particles were drawn from the posterior distribution and the model simulated for 5 years with each particle and no controls in effect, and the output of this saved. Each saved output was then continued with each control scenario, simulated for another 5 years, 100 times. To represent outside infection pressure, 10 infectious farms were seeded at random at the transition from no controls to possible controls - it was expected that this would have little effect given the large number of immune animals. In this manner, the effect of the control parameters could be isolated from the effect of the parameter values, as well as enabling the estimation of this effect conditional on the posterior.

79 unique control policy scenarios were assessed, using combinations of the control strategy parameters given in Table 3. We took as our outcomes of interest: (1) the total numbers of infected farms over the 5 year period (Σ_*s*_, eq. (5)), (2) the ***p***_***e***_**!** (***p***_***e***_**!**) (taken to be the proportion of simulations where the disease was eliminated), (3) and the average Time To Elimination (TTE) conditional on elimination having occurred. These were compared to a scenario where no control policies were implemented.

(5) $$\Sigma_{s}=\overset{F}{\underset{i}{\sum }}\overset{\mathrm{tmax}}{\underset{t=1}{\sum }}1\left(i,t\right)$$

Where *s* indicates a specific simulation output from the set of simulation outputs *O*, and |*O*| indicates the total number of simulations.

(6) $$p_{e}=\frac{1}{\left|O\right|}\overset{\left|O\right|}{\underset{s}{\sum }}\overset{F}{\underset{i}{\sum }}1\left(X_{i,tmax}^{EI}=0\right)$$

**Table 3:** **Control Policy Parameter Value Sets.** Each unique combination of these was simulated. ’-’ indicates the policy is not in effect.

Parameter	Parameter Values (unit)
Vaccine Efficacy	{65, 80, 90} %
Mass Vaccination Interval	{-, 365, 182} days
Ring Vaccination Radius	{-, 5, 10} km
Shipment Control Radius	{-, 5, 10} km

The subset of simulation outputs where eradication occurs is denoted *O*_*e*_, *O*_*e*_ ⊂ *O*, and *T*_*s*,*e*_ is the set of *t* where $X_{i,t}^{EI}=0$ for simulation *s* ∈ *O*_*e*_. TTE is then:

(7) $$TTE=\frac{1}{\left|O_{e}\right|}\overset{\left|O_{e}\right|}{\underset{s}{\sum }}min\left(T_{s,e}\right)$$

#### 2.4.1 Sensitivity analysis

To assess which parameters were most important to different control policies, sensitivity analysis was performed for combinations of the parameters in Table 3, Table 4 different combinations of control policies (abbreviated for space): /M/- for mass vaccination alone; R/−/− for ring vaccination alone; −/−/S for shipment control alone; R/M/- combined mass and ring vaccination; -/M/S combines mass vaccination and shipment control; R/-/S combines ring vaccination with shipment controls; and M/R/S combines all three policies. Latin Hypercube Sampling (LHS) was used to generate 120 samples for 12 parameters (Table 4), and simulated 5 times for 5 year with 100 samples from the joint posterior distribution.

**Table 4:** **Sensitivity Analysis Parameter Ranges.** Related parameters are grouped with each other. All parameters are drawn from a uniform distribution between the minimum and maximum value. Whether they are continuous (C) or discrete (D) is indicated in the Type column. NB; Vaccine capacity VC is generated *VC* = ⌊10^*x*^, *x* ∼ *U*(0, 3.04)⌋.

Parameter	Value Min, Max	Type
Detection Probability	0, 100 %	C
Detection Delay	1, 14 days	D
Vaccine Efficacy	0, 100 %	C
Vaccine Duration	60, 365 days	D
Vaccine Capacity	1, 1096 farms∗	D

Ring Vaccination Radius	1,10 km	C
Ring Vaccination Coverage	1100 %	C

Shipment Control Radius	1, 10 km	C
Shipment Control Duration	1, 14 days	D
Shipment Control Compliance	1, 100 %	C

Mass Vaccination Interval	60, 365 days	D
Mass Vaccination Coverage	1, 100 %	C

After simulation of the parameters, outputs were calculated and analysed. Partial Rank Correlation Coefficient (PRCC) sensitivity analysis was performed for each output to assess the importance of each parameter to the outcomes [Marino et al., 2008, Wu et al., 2013].

### 2.5 Analysis

Analysis was done in *R 4.0.5* [R Core Team, 2023]. PRCC analysis, a sensitivity analysis index, was done using the package *epiR 2.0.19* [Marino et al., 2008; Stevenson & Sergeant, 2021]. Plots were done using *ggplot2 3.3.3* and arranged using *patchwork* [Wickham, 2016; Pedersen, 2023].

## 3 Results

### 3.1 Model fit

The identifiability analysis indicated that the most identifiable parameter set was the combination of kernel *ξ* (transmission), *η* (shape), and *ν* (scale). It was this set of parameters that were used for the parameter estimation using the real data. The full results of this analysis are contained in the supplementary material.

**Fig. 4:**
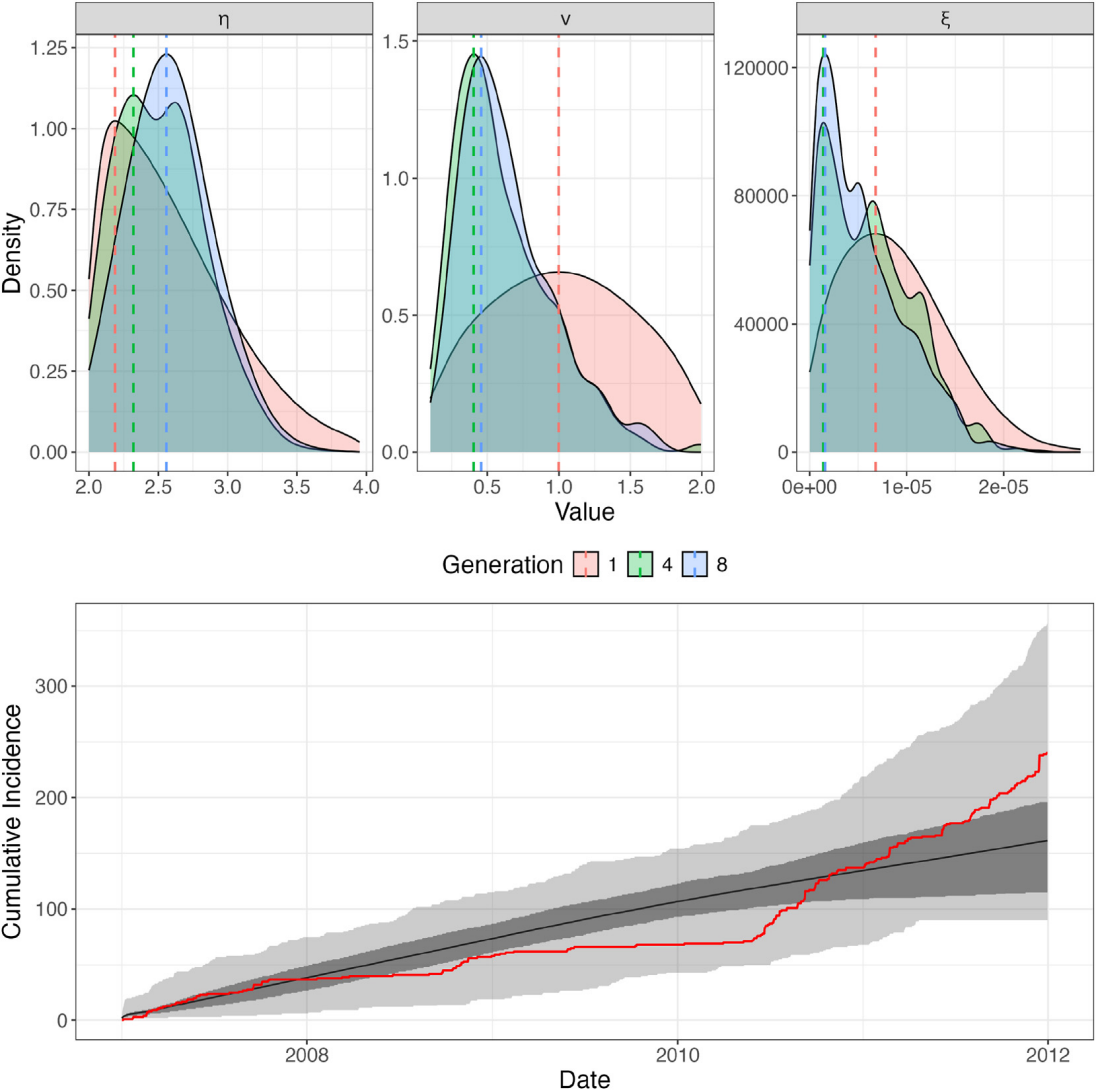
**Posterior Model Fit.** The upper panels show the marginal posterior distributions of the parameters *ν* (left panel), *η* (middle panel) and *ξ* (right panel) after 8 generations of ABC-SMC with 500 particles per generation. Each parameter demonstrates a clearly defined peak, for *ν* this peak corresponds to a value of 0.5, for *η* to 2.48, and for *ξ* to 1.43e-6. Each dotted line indicates this peak for its generation. The lower panel shows a comparison of the observed cumulative outbreak incidence (in red) with the range of the simulated incidence of the final generation (grey). The black line indicates the mean of this distribution, the darker grey area corresponds to the IQR, and the lighter grey the minimum and maximum of the simulated distribution. We see a good fit to the true data.

Using the ABC-SMC algorithm with the identified parameters, the most likely marginal parameter values were *ν* = 0.46, *η* = 2.48, and *ξ* = 1.59*e* − 6. Marginal posterior distributions of these parameters are shown in Fig. 4, demonstrating relatively tight and sharp peaks around these values.

Fig. 4 also demonstrates the good fit of the posterior simulated cumulative incidence curve with the observed curve (in red). The simulated data is a relatively tight distribution around the observed data, although it does not reflect the plateau in outbreaks around 2009-10 as well.

**Fig. 5:**
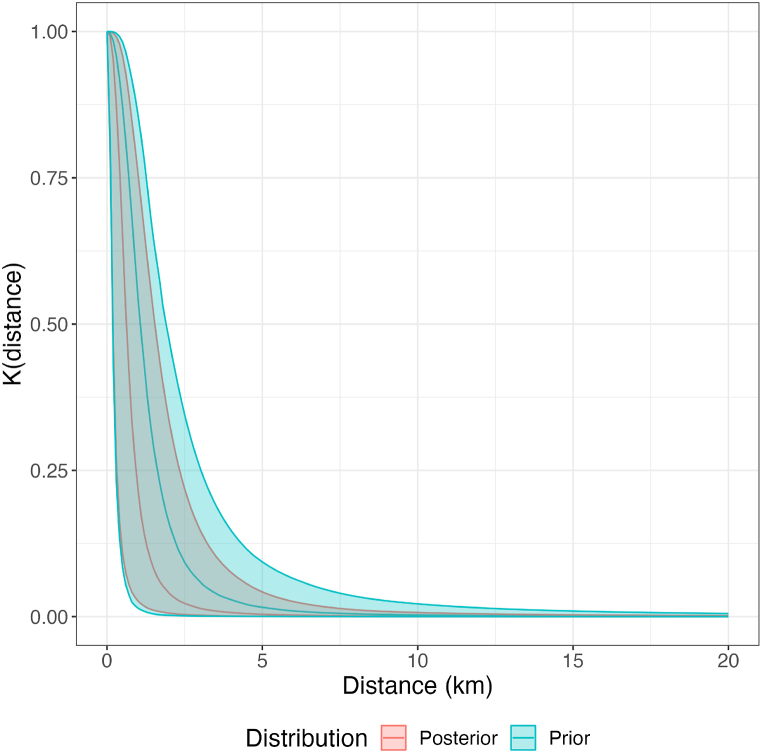
**Prior and Posterior Dispersal Kernels.** Prior and posterior kernels are displayed with their median value as a line and their 95% credible interval as their respective shaded areas. The posterior narrows the kernel credible interval, mainly by reducing the probability of higher probability of transmission.

Using our joint posterior distribution for kernel *ν* (scale) and *η* (shape) we calculate the posterior kernel with credible intervals up to a distance of 20 km, displayed in Fig. 5. The kernel using our prior distribution, is displayed for reference. The prior kernel is included in the CI of the posterior, but the posterior median is clearly lower for all distances, indicating less probability of transmission.

### 3.2 Controls

**Fig. 6:**
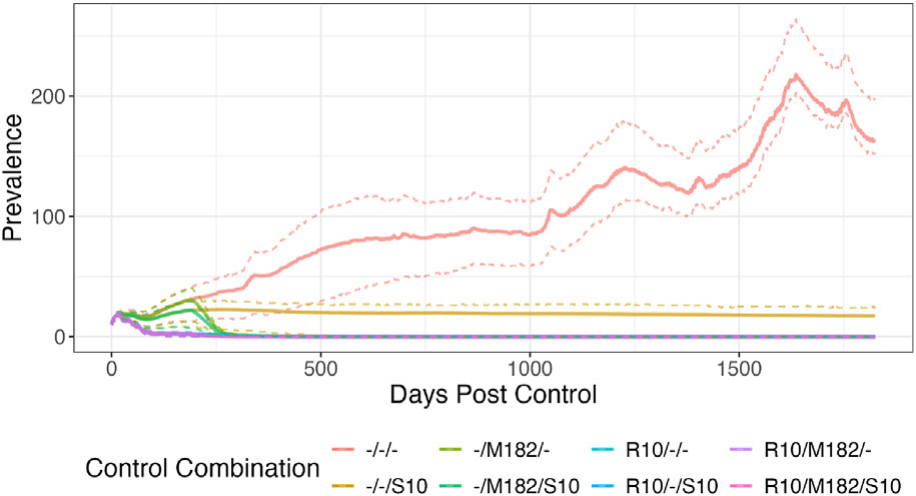
**Simulated Effect of Different Combinations of Control Policies.** The simulated prevalence (number of farms infected) after controls were or were not implemented.To make the main comparisons clearer, only maximum efficacy versions of each policy are included here (i.e. M182 indicates mass vaccination at an interval of 182 days, R10 and S10 a radius of 10 km). Coloured lines indicates mean prevalence for a policy combination. Areas bounded by the same-colour dotted line above and below indicate the IQR of the simulated prevalence.

Fig. 6 shows the simulated post-control prevalence of FMD-infected farms given a (maximum efficacy) policy combination. With no controls the posterior average prevalence increases slowly over time to a peak of just over 200 farms infected, before beginning to decline again.

In comparison, all three controls avert this increase. Shipment controls (alone) will at minimum hold prevalence to approximately the same level as at the beginning of the period (averaging approximately 1 outbreak every 5 days), albeit trending slightly downwards. Mass vaccination absent ring vaccination can lead to local eradication within a year; the addition of ring vaccination allows the first vaccination campaign to achieve local eradication within a few months.

### 3.3 Sensitivity analysis

**Fig. 7:**
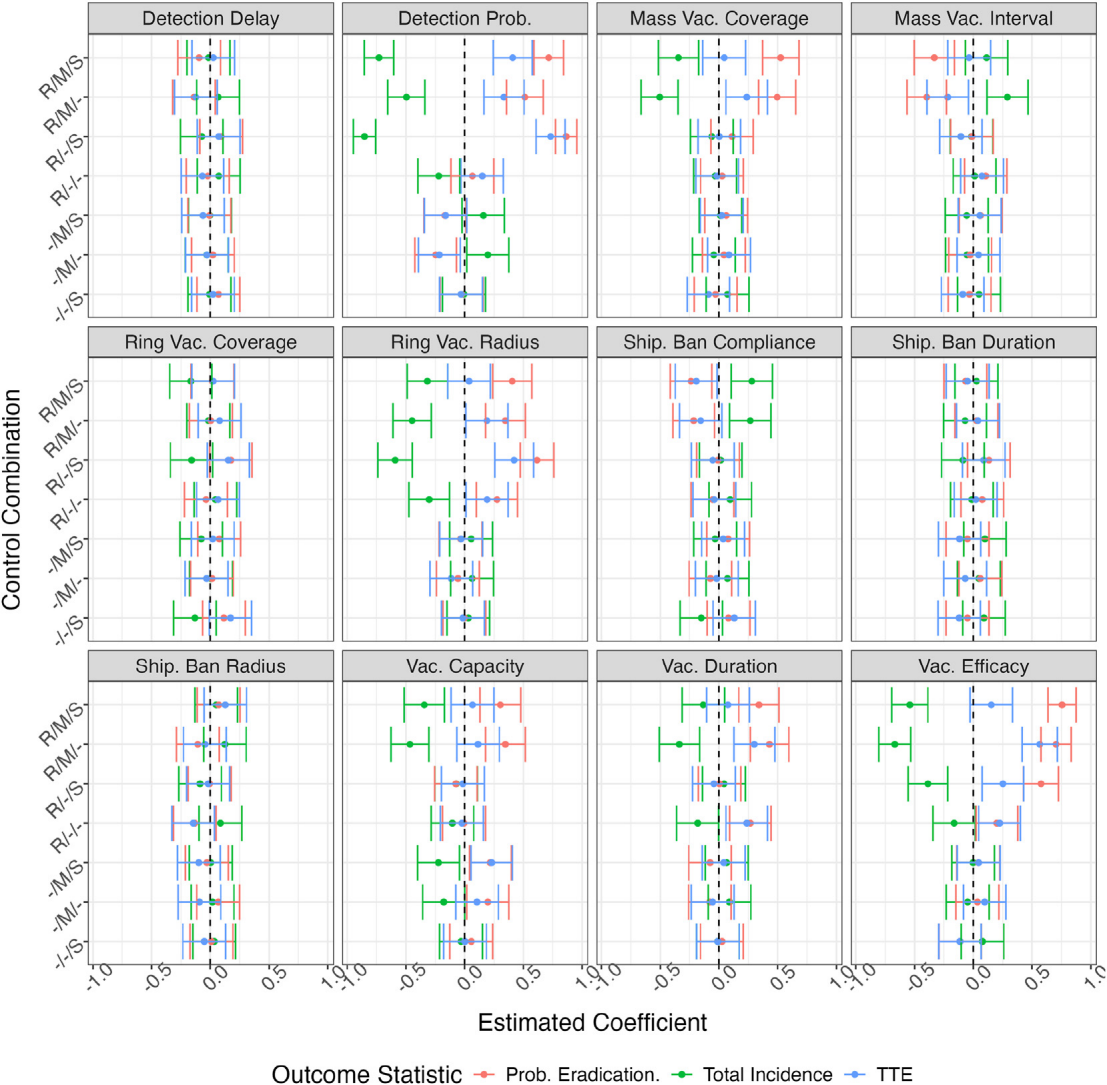
**Estimated Correlations of Policy Parameters.** The estimated correlations between control policy parameters and either total incidence (green), probability of eradication *p*_*e*_ (red), or time to eradication TTE (blue). Each panel corresponds to a policy parameter, and each row in each panel is the correlations for a specific combination of control policies indicated using ”—” format (e.g. ”-MS” indicates mass vaccination M and shipment control S are in effect, but not ring vaccination R). Error bars denote the 95% confidence interval. Dotted lines are shown at 0 for ease of analysis.

Sensitivity analysis focused on three different outputs: average total incidence (Σ_*s*_), the probability of elimination (*p*_*e*_) and the time to elimination (TTE) conditional on elimination. The coefficients for these are displayed in Fig. 7.

For surveillance, detection delay was not correlated with any of these outcomes (confidence intervals overlap 0). However, for scenarios involving ring vaccination R, the probability of detection was strongly negatively correlated with total incidence and positively correlated with *p*_*e*_ and TTE.

Mass vaccination coverage and interval were negatively correlated with the total incidence and positively correlated with *p*_*e*_; coverage was positively associated with TTE when mass vaccination was implemented with ring vaccination, but no association was seen when shipment controls were included as a policy option. In the opposite direction, increasing the mass vaccination interval (longer times between pulses) was associated with a lower probability of eradication and more infected farms, but had little effect on the time to eradication.

For ring vaccination, the most important parameter in this analysis is the radius of the ring, which is strongly correlated with the output variables. Increasing the radius was associated with a greater probability of eradication in a shorter time, and lower numbers of infected farms overall. However, the coverage achieved was less correlated with outcomes, most coefficient point estimates remaining in the vicinity of 0.

The parameters of the shipment control policy option were not strongly correlated with any of the outcomes, with most coefficients hovering around 0.

For the vaccine itself, vaccine capacity had no effect on time to eradication but was positively correlated with *p*_*e*_ and negatively correlated with total incidence. Vaccine duration was little correlated with outcomes in most scenarios, with the greatest coefficients seen when implementing mass vaccination with ring vaccination; the addition of shipment controls to the control policy mix reduced correlation coefficients for ring and mass vaccination. Finally, for vaccine efficacy there is are increasingly strong correlations in all control policy combinations that include ring vaccination, with the expected negative correlation with total incidence and positive correlation for *p*_*e*_.

## 4 Discussion

The model provides a reasonable fit to the observed incidence in Erzurum province, 2007–2012, with most simulations in the final generation of fitting. The most likely values from the posterior estimates indicate a significant narrowing of the dispersal kernel - transmission was more local than our prior beliefs; the overall farm-to-farm force of infection appears to be lower than in the UK. This may be related to climatic variability; Turkey's climate is hotter on average than the UK's, and the virus does not survive as long in hotter temperatures [Mielke & Garabed, 2020].

Our results demonstrate clearly that although all three control policies are effective in achieving control of disease spread, reactive ring and prophylactive mass vaccination are essential to maximise the probability eradication is achieved. This agrees both with the literature and common sense; mass vaccination is clearly responsible for the current FMD status of most of Europe (free without vaccination) free of FMD status of most of Europe as well as South America (free with vaccination) [Leforban, 1999; Paton et al., 2009; Parida, 2009; Naranjo & Cosivi, 2013]. The discrepancy between the decades required for elimination in reality and the predicted 1–2 years in our simulations is likely due to the much smaller area simulated and the lack of outside re-introductions of disease. Ring vaccination is also a well established reactive control measure, used in many countries and theoretically well-suited to controlling the spread of FMD [Tildesley et al., 2006; Pluimers et al., 2002; Muleme et al., 2012].

Shipment controls in our model also provide a useful policy tool to reduce but not eliminate the spread of FMD. As seen in Fig. 6, the effect of shipment controls was to reduce the spread of the disease, preventing prevalence from increasing but not driving it to eradication. The movement of infected animals is considered a key transmission route of FMD, and previous work [Tildesley et al., 2019] has found that banning the movement of livestock in a small area surrounding a farm was the optimal control response for an epidemic of FMD. However, the role of movement restrictions in preventing the spread of FMD may be obviated in a situation of endemic disease as the disease is already seeded in many different locations; the effect of restricting one or another farm in their ability to move livestock legally, then, would be much reduced. Supporting the limited role of livestock movements in endemic FMD, previous work has found that they were not involved in the maintenance of endemic FMD [Di Nardo et al., 2011; Schnell, 2019; Guyver-Fletcher et al., 2022]. In summary, shipment control policies are useful in epidemics and in concert with other policy tools, but cannot be relied upon alone to drive eradication.

The efficacy of mass vaccination means it is little surprise all variables associated with it are (in our sensitivity analysis) correlated with lower incidence and more likely eradication. In the same manner, the inefficacy of shipment controls (S) in driving eradication leads to shipment-related parameters having limited effect. Ring vaccination, however, is split; the coverage of farms in the ring around an infected farms appears much less correlated with reduced incidence than the radius of the ring itself. It may be that this is necessary to contain the spread or that closer farms are likely already infected - it is ineffective to vaccinate all nearby farms if they are already infected or the disease has ’jumped’ past them.

The delay to detection having little effect on the outcomes is more surprising; these results suggest it is more important to ensure detection of an infected farm (P(detect)) than it is to invest in making the detection promptly. It may be that the fit transmission values do not represent enough risk of onward transmission, such that 14 days is almost always adequate. Alternatively ring vaccination may be effective enough that, again, 14 days is adequate.

Finally, the vaccine parameters we investigated span the range from strongly correlated with the outputs of interest (vaccine efficacy), to low-moderately correlated (capacity & duration). A longer duration FMDV vaccine has long been a goal, with ongoing research efforts [Singh et al., 2019; Kamel et al., 2019]; our results suggest that these are substitutable with thoroughly implemented control policies.

This model is not perfect and retains some limitations. For example, we assume the circulation of only one FMDV serotype due to lack of data despite there being evidence of differing transmission dynamics [Pacheco et al., 2012; Pacheco et al., 2016]. Additionally, carrier animals are common in endemic regions, with as many as 50% of naïve cattle becoming carriers after exposure [Stenfeldt & Arzt, 2020]. Although there is fierce debate as to whether carrier animals can infect naïve animals [Guyver-Fletcher et al., 2022], the presence of such large numbers of animals with constantly stimulated immune systems can scarcely fail to have an impact on disease dynamics. We believe this is an important direction for future work in this area. However, we remain confident about the value of this modelling framework. Due to our endemic focus, this work does include factors specific to endemic FMD, such as interference from maternally derived antibodies and waning natural and vaccine-derived immunity. As outlined in [Zaheer et al., 2020], the majority of disease compartmental models of FMD do not consider such factors, and a model that does should more accurately capture some of the dynamics of the endemic disease state. Additionally, the model allows for the provision of routine prophylactic vaccination (mass vaccination), a control policy routinely ignored by models attempting to model an epidemic of FMD in a free country due to the unlikelihood of its implementation. Finally, the framework is capable of good fits to high-quality real-world data from an endemic setting.

Our work suggests that most if not all of the control of FMD can be achieved by focusing on comprehensive surveillance of farms, and high coverage ring and mass vaccination campaigns. This may be a boon to countries wishing to control the disease, as it likely much easier politically to implement than movement restrictions, which are often unpopular with farmers and can cause direct economic losses. Movement restrictions can also incur high monitoring costs to ensure compliance, whereas vaccination only requires occasional visits from a local veterinary professional. This work provides support for the use of vaccination as a control policy, and suggests that the use of movement restrictions should be carefully considered in endemic situations.

This work has developed a spatially-explicit metapopulation model of the spread of endemic FMD and estimated spatial transmission parameters using high-quality data from the Republic of Turkey. This work also provides a fitted dispersal kernel estimated on endemic data, to our knowledge the first. We use it to assess the three main control methods used within Turkey, alone and in combination, for their efficacy at reducing incidence and probability of achieving eradication. Finally, sensitivity analysis of control parameters provided estimates of the importance of specific aspects of each control policy.

This works shows that ring vaccination and mass vaccination are the most effective control policies for regions where FMD is endemic that are aiming for eradication; controls on livestock movements in an endemic situation are useful but cannot drive eradication on their own. Ring vaccination at a radius of 10 km, in combination with biannual mass vaccination and livestock shipment controls, emerged as the clear optimal policy (of those assessed) for reducing the circulation of disease and maximising the probability of elimination. When considering these policies, it was most important to maximise the radius of ring vaccination and mass vaccination coverage, and minimise the interval mass vaccination is implemented on. More research to bound the effects of these assumptions on policy effectiveness should be undertaken.

## CRediT authorship contribution statement

**Glen Guyver-Fletcher:** Writing – review & editing, Writing – original draft, Visualization, Validation, Software, Resources, Project administration, Methodology, Investigation, Formal analysis, Data curation, Conceptualization. **Erin E. Gorsich:** Writing – review & editing, Supervision, Project administration, Methodology, Funding acquisition, Conceptualization. **Chris Jewell:** Writing – review & editing, Methodology. **Michael J. Tildesley:** Writing – review & editing, Supervision, Project administration, Methodology, Funding acquisition, Conceptualization.

## Declaration of competing interest

The authors declare that they have no known competing financial interests or personal relationships that could have appeared to influence the work reported in this paper.
