# Supplementary material for: Controlling endemic foot-and-mouth disease: Vaccination is more important than movement bans. A simulation study in the Republic of Turkey
Source: Infect Dis Model. 2025 Feb 13;10(2):702–15. doi: 10.1016/j.idm.2025.02.006 (PMC11907466; doi:10.1016/j.idm.2025.02.006)
Supplement: Multimedia component 1 [file mmc1.pdf]

8 Supplementary Material

Table S1: Mean outcomes of control policy combinations.

	VE90			VE65			VE80		
Control	Σ_s	p_e	TTE	Σ_s	p_e	TTE	Σ_s	p_e	TTE
-/-/-	1824	0.10	391	1824	0.10	391	1824	0.10	391
R5/-/-	135	0.79	466	163	0.75	500	143	0.78	483
R10/-/-	28	1.00	302	31	1.00	362	29	1.00	311
-/-/S5	343	0.57	717	344	0.58	731	332	0.58	699
R5/-/S5	30	1.00	354	32	0.99	379	30	1.00	356
R10/-/S5	21	1.00	227	23	1.00	263	22	1.00	233
-/-/S10	327	0.58	724	321	0.61	714	320	0.61	705
R5/-/S10	29	1.00	348	32	1.00	385	30	1.00	358
R10/-/S10	22	1.00	226	23	1.00	262	22	1.00	231
-/M182/-	69	1.00	328	71	1.00	394	71	1.00	342
R5/M182/-	32	1.00	260	33	1.00	293	32	1.00	265
R10/M182/-	25	1.00	216	26	1.00	246	25	1.00	219
-/M182/S5	53	1.00	307	54	1.00	356	53	1.00	314
R5/M182/S5	25	1.00	234	27	1.00	264	26	1.00	241
R10/M182/S5	21	1.00	196	22	1.00	219	22	1.00	197
-/M182/S10	53	1.00	309	54	1.00	357	53	1.00	315
R5/M182/S10	25	1.00	235	26	1.00	264	25	1.00	240
R10/M182/S10	21	1.00	192	22	1.00	217	21	1.00	199
-/M365/-	162	1.00	576	208	0.92	777	165	1.00	607
R5/M365/-	41	1.00	373	46	1.00	437	42	1.00	382
R10/M365/-	27	1.00	268	28	1.00	304	27	1.00	273
-/M365/S5	88	1.00	468	95	1.00	563	91	1.00	488
R5/M365/S5	27	1.00	301	29	1.00	327	27	1.00	305
R10/M365/S5	22	1.00	220	23	1.00	252	22	1.00	224
/M365/S10	89	1.00	473	93	0.99	556	88	1.00	479
R5/M365/S10	27	1.00	306	29	1.00	326	27	1.00	304
R10/M365/S10	21	1.00	216	23	1.00	253	22	1.00	229

Table S1 shows the values of the policy outcomes from the control policy combination analyses. The scenario with no control averages 1,824 infected farms across the simulated period, with a probability of spontaneous eradication of approximately 0.1 and a time to eradication of 391 (361, 421). The addition of controls immediately reduces the observed incidence, and the probability of eradication increases to a minimum of 0.55. Further increases in control intensity make eradication almost certain.

This is likely a consequence of, after 5 years of no controls, the majority of farms having some latent immunity (compare to sensitivity analysis outcomes below) - in addition to the lack of outside reintroduction. The absolute values of the outcomes are not necessarily informative, however, the relative comparisons between policy options are informative as these are retained in the two analyses.

8.1 Sensitivity Analysis Outcomes

Table S2: Summary of Mean Outcomes by Control Policy for PRCC. The policy outcomes for each control policy combination, results from PRCC analysis. Means provided, 95% confidence intervals in brackets.

**Control**	**Total Incidence** (Σ_s)	**Prob. Eradication** (p_e)	**TTE**
-/-/-	$1,774$ $(1,763,\ 1,786)$	0.11 (0.10, 0.11)	408, (402, 414)
-/-/S	$1,081$ $(1,070,\ 1,092)$	0.15 (0.14, 0.15)	740, (728, 753)
-/M/-	$1,213$ $(1,201,\ 1,225)$	0.32 (0.31, 0.33)	$1,072$, $(1,060,\ 1,084)$
-/M/S	809 (800, 818)	0.46 (0.45, 0.47)	$1,188$, $(1,179,\ 1,198)$
R/-/-	$1,184$ $(1,173,\ 1,195)$	0.18 (0.17, 0.18)	845, (832, 857)
R/-/S	834 (824, 843)	0.43 (0.43, 0.44)	$1,210$, $(1,201,\ 1,220)$
R/M/-	951 (941, 961)	0.42 (0.41, 0.42)	$1,145$, $(1,135,\ 1,155)$
R/M/S	722 (714, 730)	0.61 (0.60, 0.61)	$1,221$, $(1,214,\ 1,228)$

As the sensitivity analysis looked at several different control policy combinations, sampling over the posterior kernel distribution, we can calculate policy outcomes for these. Table S2 shows the policy outcomes for each control combination used - there is a clear decrease in the total incidence over the period with increasing control efforts, and an increase in the probability of eradication and time to eradication.

Two things should be noted: the sensitivity analysis does not run the model to an endemic state before implementing controls, so most of the population is still susceptible - this should increase total incidence and decrease probability of eradication; second, these results average over both the

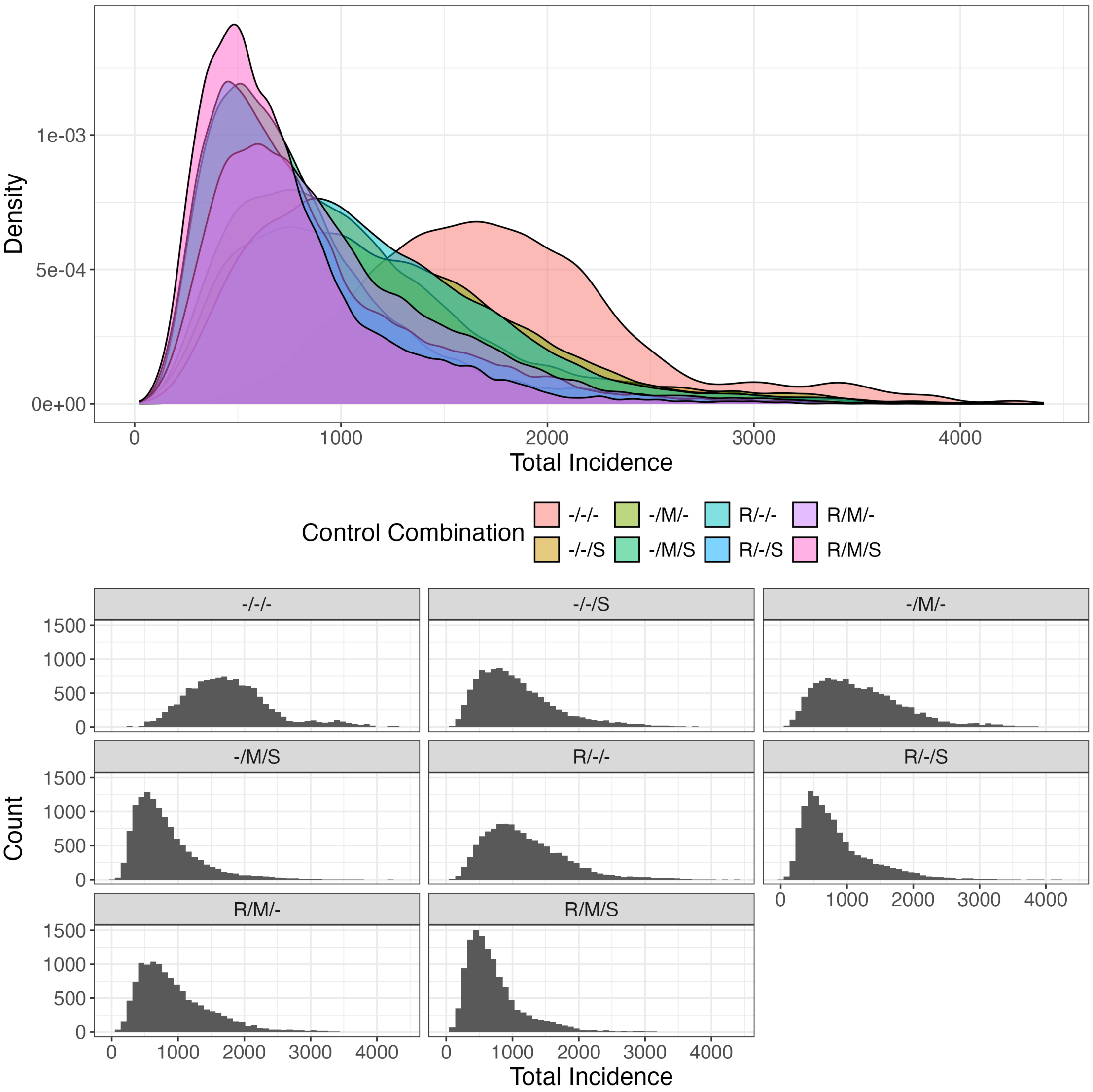

Density
1e-03
5e-04
0e+00
0
1000
2000
3000
4000
Total Incidence
Control Combination
-/-/-
-/M/-
R/-/-
R/M/-
-/-/S
-/M/S
R/-/S
R/M/S
Count
1500
1000
500
0
Total Incidence

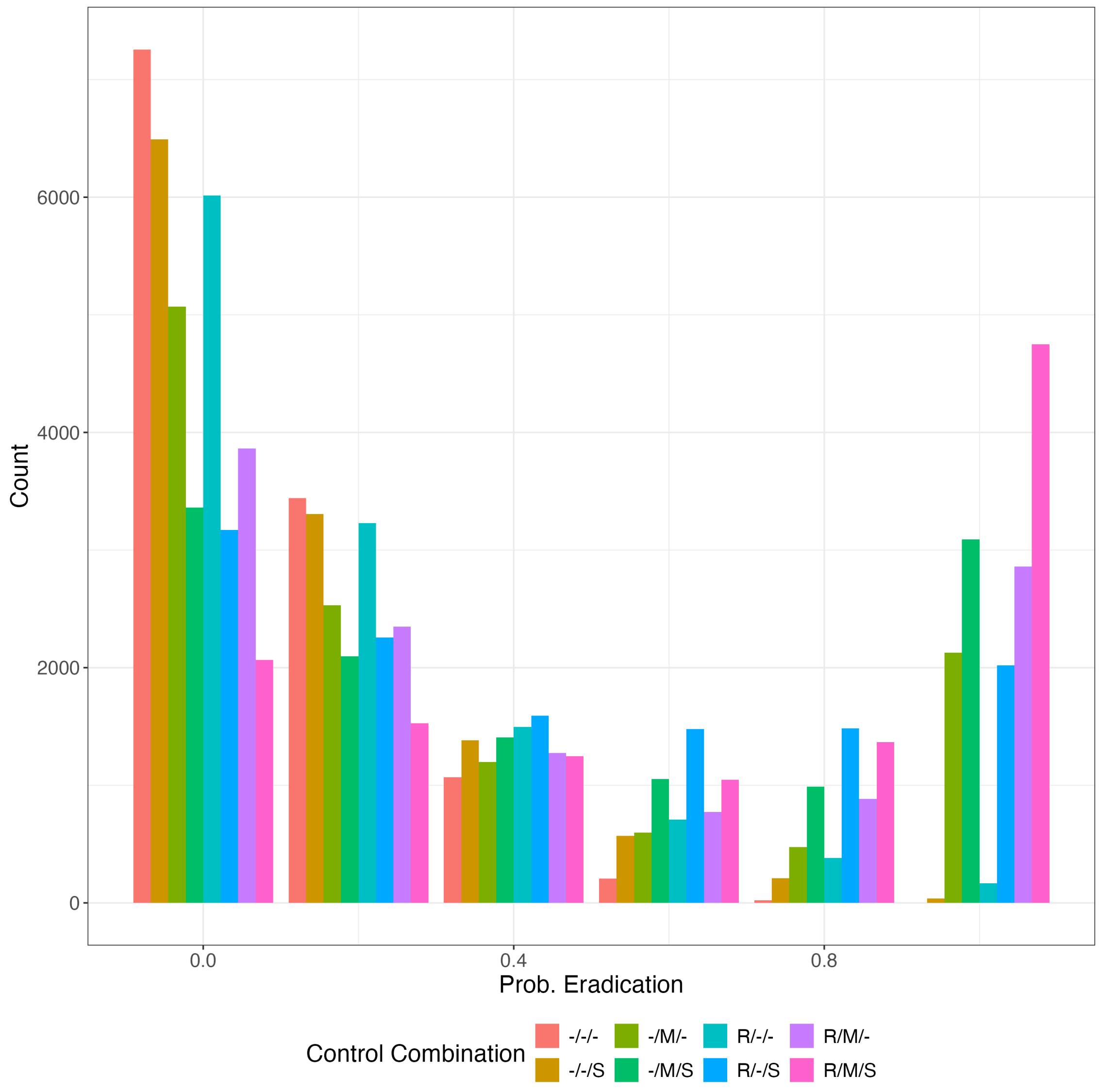

Count
6000
4000
2000
0
0.0
0.4
0.8
Prob. Eradication
Control Combination
-/-/-
-/-/S
-/M/-
-/M/S
R/-/-
R/-/S
R/M/-
R/M/S

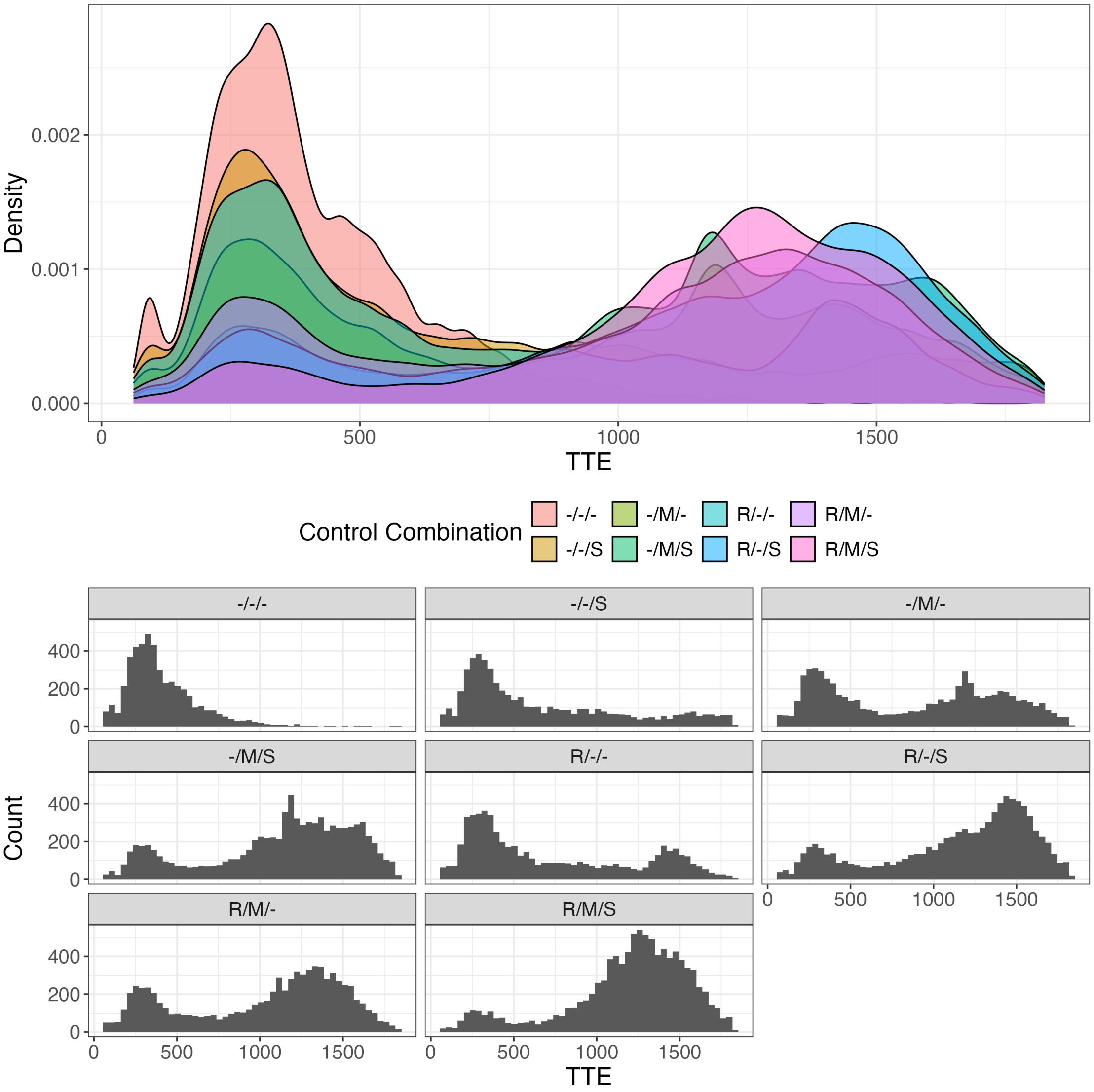

Density
0.000
0.001
0.002
0
500
1000
1500
TTE
Control Combination
-/-/-
-/-/S
-/M/-
-/M/S
R/-/-
R/-/S
R/M/-
R/M/S
Count
400
200
0
TTE

posterior kernel parameters and the varying parameters of the sensitivity analysis - they therefore include simulations with, for example, very low detection rates or vaccine efficacy. Both of these factors would lead to lower estimates of efficacy.

There is a slightly paradoxical result where TTE is positively correlated with the probability of eradication - i.e. as eradication becomes more certain the time to achieve it lengthens. This is a result of increasing control intensity 'converting' simulations where eradication would not have been achieved in the 5 years to simulations where they are eradicated, but slowly. For example, at low control intensity the disease either dies out fast or remains endemic, but scenarios with more control effort include simulations where the disease is gradually eradicated. Hence, the mean TTE increases even as the probability of eradication also increases.

8.2 Identifiability Analysis

Identifiability analysis was done using ABC-SMC with 500 particles per generation, using a weighted multivariate normal distribution for perturbation, and the same distance metric computed on the simulated incidence compared to the "true" incidence. The aim of this was to recover the original sampled parameter value for as many of the parameters as possible, and to find as large a set of parameters as possible that were identifiable in this manner.

8.2.1 Parameters and Prior Distributions

Table S3: The prior distributions used to generate "true" values for parameter identifiability analysis.

Parameter	Parameter Description	Distribution
β	Acutely infectious transmission	$U(0.135, 1.8)$
ϕ_R^{-1}	Average duration of recovered state	$U(150, 550)$
ξ	Inter-farm per capita transmission	$N(6.8e-6, 6.8e-6)$
ν	Kernel scale parameter	$N(1.0, 0.75)$
η	Kernel shape parameter	$N(2.0, 0.75)$

This was first done for the parameters within-farm transmission β, the duration of immunity ϕ_R^{-1}, the between-farm per-capita transmission parameter ξ, and the kernel ν and kernel η parameters, this set of parameters is labelled **ID1**. Subsequent to the results of this, another attempt was made dropping the kernel η parameters (labelled **ID2**), and then another attempt was made also dropping the kernel ν parameter (labelled **ID3**) to try and find the optimal combination of parameters that were identifiable. The prior distributions that were sampled from are described in Table S3. A summary of the parameters used for these attempts are laid out in Table S4, and the values used for the other fixed parameters are shown in Table 1.

Table S4: The parameter sets used for the attempts to assess parameter identifiability. Each one has a set label which identifies it for ease of analysis.

Set Label	Parameter Set
**ID1**	$\{\beta, \lambda, \xi, \nu, \eta\}$
**ID2**	$\{\beta, \lambda, \xi, \nu\}$
**ID3**	$\{\beta, \lambda, \xi\}$
**ID4-2-1**	$\{\beta, \xi\}$
**ID4-2-2**	$\{\beta, \nu\}$
**ID4-2-3**	$\{\beta, \eta\}$
**ID4-2-4**	$\{\nu, \xi\}$
**ID4-2-5**	$\{\eta, \xi\}$
**ID4-2-6**	$\{\nu, \eta\}$
**ID4-3-1**	$\{\beta, \nu, \xi\}$
**ID4-3-2**	$\{\beta, \nu, \eta\}$
**ID4-3-3**	$\{\nu, \eta, \xi\}$
**ID4-3-4**	$\{\beta, \eta, \xi\}$

Finally, each possible 2-parameter or 3-parameter combination of the parameters β, ξ, ν, and η were assessed in turn using the same procedure as described previously. ϕ_R^{-1} was no longer assessed due to the results of the previous attempts, and reverted to the value described in Table 1. The specific parameter combinations are shown in Table S4.

A note: the figures included in this refer to $kernel\_scale, kernel\_shape$, and $transmission$ due to a notation change. $kernel\_scale = \nu$, $kernel\_shape = \eta$, $transmission = \xi$.

8.2.2 Results

Using the parameter set outlined in Table S4 for **ID1** for identifiability analysis, the most identifiable parameter analysed was transmission ξ, which was recovered 3 out 5 times. Recovery of a parameter was decided based on whether the highest peak of the posterior distribution was close to the "true" value, and whether the posterior probability distribution was significantly different to the prior distribution. The final density plots of each parameter are shown in Figure S1. In this figure parameter recovery is taken as when the peak (assuming there is one) of the density plot is at, or close to, the "true" value indicated by the vertical black lines.

The other parameters analysed were either recovered once out of the 5 attempts, in the case of β, ν, and η, or not recovered at all in the case of ϕ_R^{-1}. It was considered that perhaps there were too many parameters and the parameter space was too large.

Subsequent to this attempt, which of the parameters of the set identified by the label **ID2** were attempted, shown in Figure S10. Performance improved with this attempt, as β was recovered 3 out of 5 times, and ν 2 out of 5 times. However, ξ was only recovered 2 out of 5 times, a reduction from the 3 seen previously. Additionally, ϕ_R^{-1} was again not recovered, with the final distribution being similar to the prior uniform distribution in all five attempts.

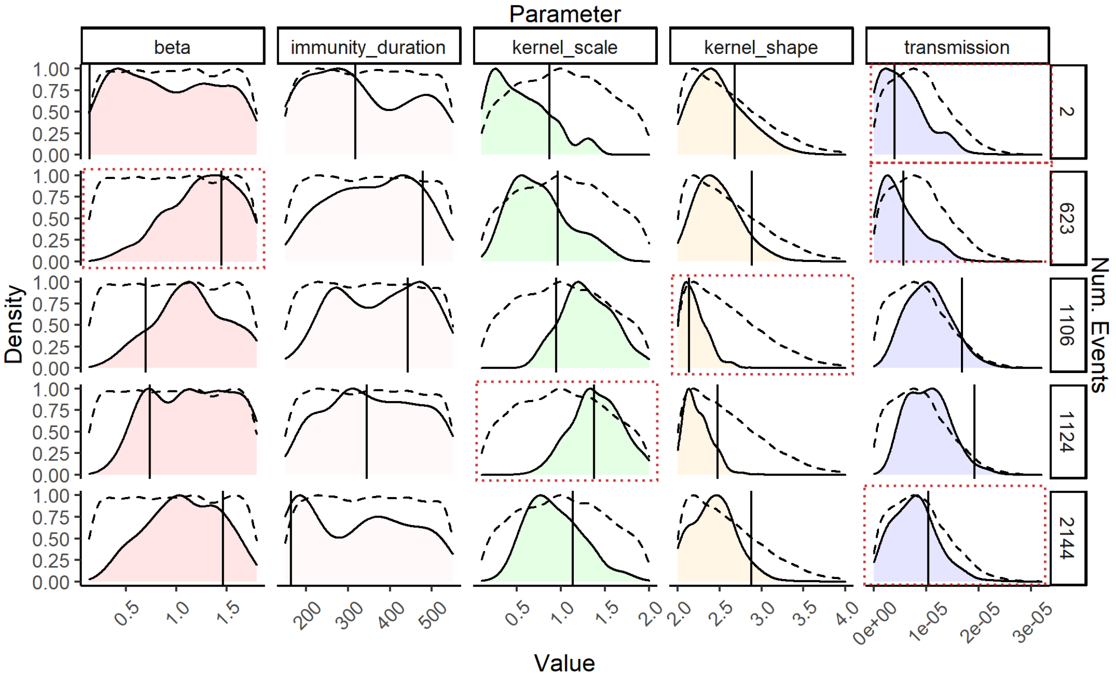

Figure S1: The density distributions of the parameter set $\{\beta, \lambda, \xi, \nu, \eta\}$ (**ID1**) after 6 generations of ABC-SMC. Each column is a different parameter, with colour-coded density plots. Each row corresponds to a different "true" value sampled from the prior distribution and recovery attempted via ABC-SMC. The black dotted line on each plot indicates the prior distribution for that parameter, and the vertical solid black line indicates the "true" sampled value for that particular parameter and fit. On the right-hand axis, the number indicate how many events were seen in that "true" outbreak, calculated as the total incidence of infection over the simulation. Higher totals indicate more information to fit to. Attempts where a parameter was considered recovered are surrounded by a dotted red box. With this set of parameters, β was recovered 1 out of 5 times, ϕ_R^{-1} was not recovered, ν was recovered 1 out of 5 times, η was recovered 1 out of 5 times, and ξ was recovered 3 out of 5 times.

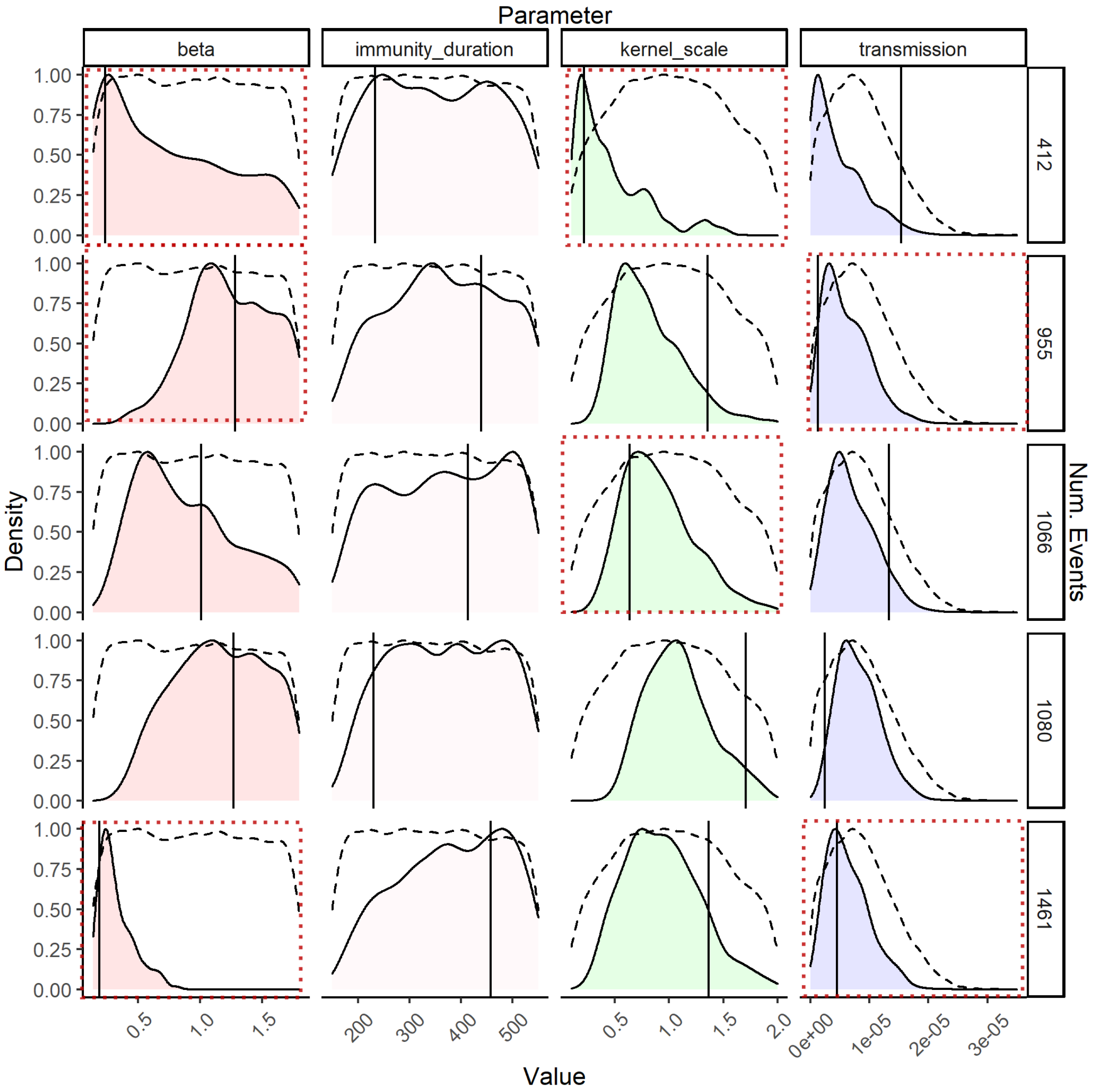

Figure S2: The density distributions of the parameter set $\{\beta, \lambda, \xi, \nu\}$ (**ID2**) after 7 generations of ABC-SMC. Each column is a different parameter, with colour-coded density plots. Each row corresponds to a different "true" value sampled from the prior distribution and recovery attempted via ABC-SMC. The black dotted line on each plot indicates the prior distribution for that parameter, and the vertical solid black line indicates the "true" sampled value for that particular parameter and fit. On the right-hand axis, the number indicate how many events were seen in that "true" outbreak, calculated as the total incidence of infection over the simulation. Higher totals indicate more information to fit to.Attempts where a parameter was considered recovered are surrounded by a dotted red box. With this set of parameters, β was recovered 3 out of 5 times, ϕ_R^{-1} was not recovered, ν was recovered 2 out of 5 times, and ξ was recovered 2 out of 5 times.

The final parameter distributions of **ID3** are shown in Figure S3. This shows an improvement in the identifiability of β, with 4 out of 5 attempts succeeding and showing tighter distributions where recovery occurred. ξ improved back to being recovered 3 out of the 5 attempts. However, ϕ_R^{-1} once again failed to be recovered, with posterior distributions similar to the prior.

As ϕ_R^{-1} had failed to be identified on 3 separate occasions, this parameter was dropped from subsequent attempts and reverted to the value established in Table 1.

Figures S4, S5, S6, S7, S8, and S9 show the results of the 2-parameter combinations of β, ξ, ν, and η identified in Table S4.

For parameter set **ID4-2-1** (fig. S4), which looked at β and ξ, performance was poor. β was recovered three times, and ξ only recovered twice. Similar performance was observed for set **ID4-2-2** (fig. S5), where β was also only recovered once, and ν was recovered 2 out of 5 times.

Parameter set **ID4-2-3** (fig. S6) assess β and η together, seeing poor performance also. β was recovered 3 out of 5 times, and η 3 out of 5.

For parameter set **ID4-2-4** (fig. S7), both ν and ξ were recovered 4 out of 5 times. This parameter set performed the best out of the 2-parameter combinations assessed.

Worst performing was parameter set **ID4-2-5** (fig. S8), where η and ξ were both only recovered once out of 5 attempts. Finally, parameter set **ID4-2-6** (fig. S9), showed ν recovered once and η recovered twice out of the 5 attempts with that set of parameters.

For the 3-parameter sets described in Table S4, set **ID4-3-1** (fig. S10) exhibited poor performance. Of the 5 fitting attempts, β was recovered 3 times, ν once, and ξ twice.

The worst performance was seen with the set **ID4-3-2** (fig. S11), where β, ν, and η were all recovered once out of the 5 attempts.

Much better performance was seen with set **ID4-3-3** (fig. S12). This assessed ν, η, and ξ together, and the least identifiable parameter with this set of parameters was η, which was only recovered 3 out of the 5 attempts. Although no parameter was recovered every time, both ν and ξ were recovered 4 out of the 5 attempts.

Finally, set **ID4-3-4** (fig. S13) demonstrated decent performance, with β, η, and ξ all being recovered on 3 out of the 5 attempts.

The parameter set that demonstrated the greatest identifiability with the greatest number of parameters was parameter set **ID4-3-3**, which assessed dispersal kernel ν, η, and ξ together. It was this set of parameters that were used for the parameter estimation using the real data.

For the 3-parameter sets described in Table S4, set **ID4-3-1** (fig. S10) exhibited poor performance. Of the 5 fitting attempts, β was recovered 3 times, ν once, and ξ twice.

The worst performance was seen with the set **ID4-3-2** (fig. S11), where β, ν, and η were all

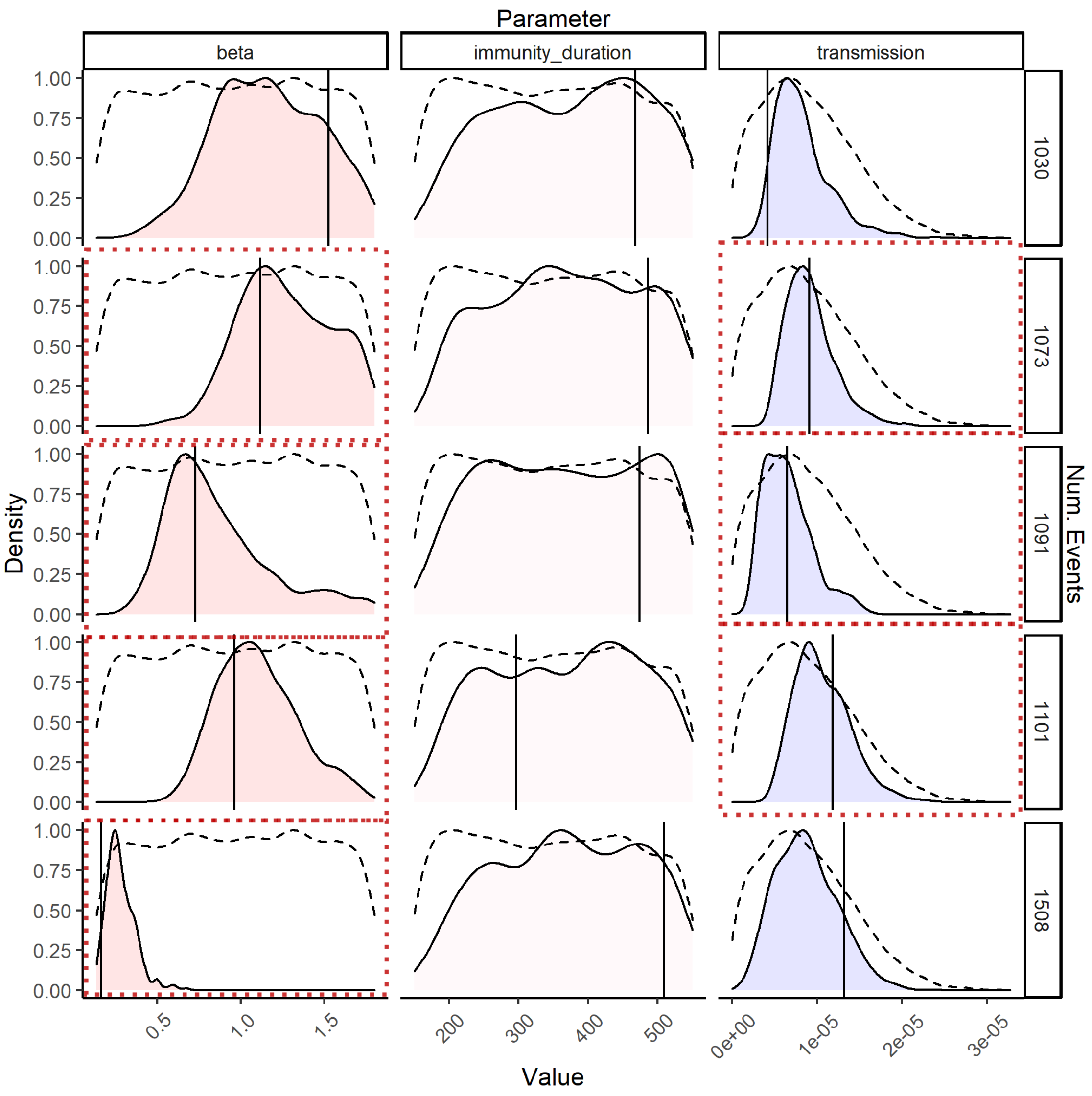

Figure S3: The density distributions of the parameter set $\{\beta, \lambda, \xi\}$ (**ID3**) after 7 generations of ABC-SMC. Each column is a different parameter, with colour-coded density plots. Each row corresponds to a different "true" value sampled from the prior distribution and recovery attempted via ABC-SMC. The black dotted line on each plot indicates the prior distribution for that parameter, and the vertical solid black line indicates the "true" sampled value for that particular parameter and fit. On the right-hand axis, the number indicate how many events were seen in that "true" outbreak, calculated as the total incidence of infection over the simulation. Higher totals indicate more information to fit to. Attempts where a parameter was considered recovered are surrounded by a dotted red box. With this set of parameters, β was recovered 4 out of 5 times, ϕ_R^{-1} was not recovered, and ξ was recovered 3 out of 5 times.

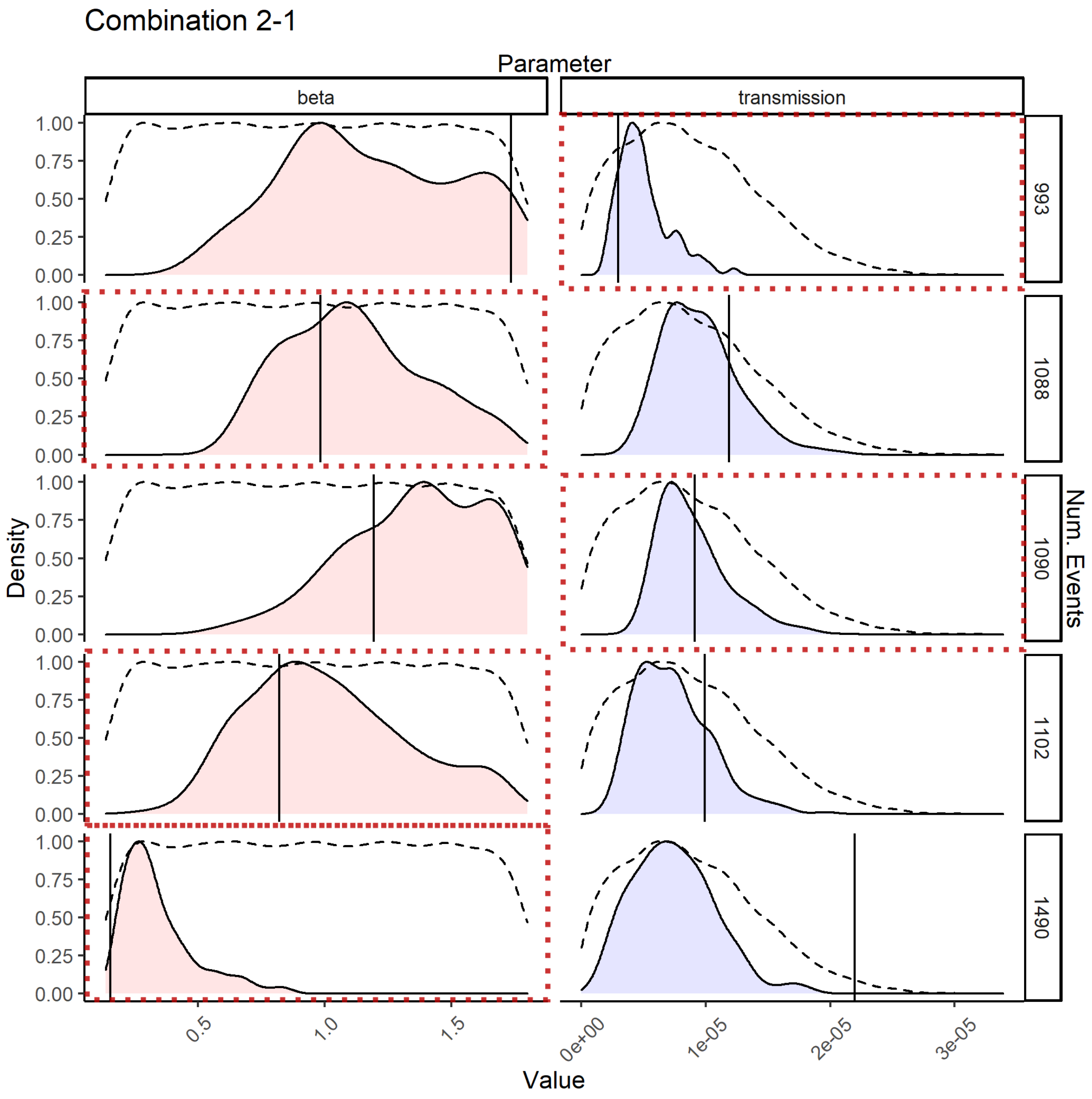

Figure S4: The density distributions of the parameters β and ξ (**ID4-2-1**) after 6 generations of ABC-SMC. Each column is a different parameter, with colour-coded density plots. Each row corresponds to a different "true" value sampled from the prior distribution and recovery attempted via ABC-SMC. The black dotted line on each plot indicates the prior distribution for that parameter, and the vertical solid black line indicates the "true" sampled value for that particular parameter and fit. On the right-hand axis, the number indicate how many events were seen in that "true" outbreak, calculated as the total incidence of infection over the simulation. Higher totals indicate more information to fit to. Attempts where a parameter was considered recovered are surrounded by a dotted red box. With this set of parameters, β was recovered 3 out of 5 times, and ξ was recovered 2 out of 5 times.

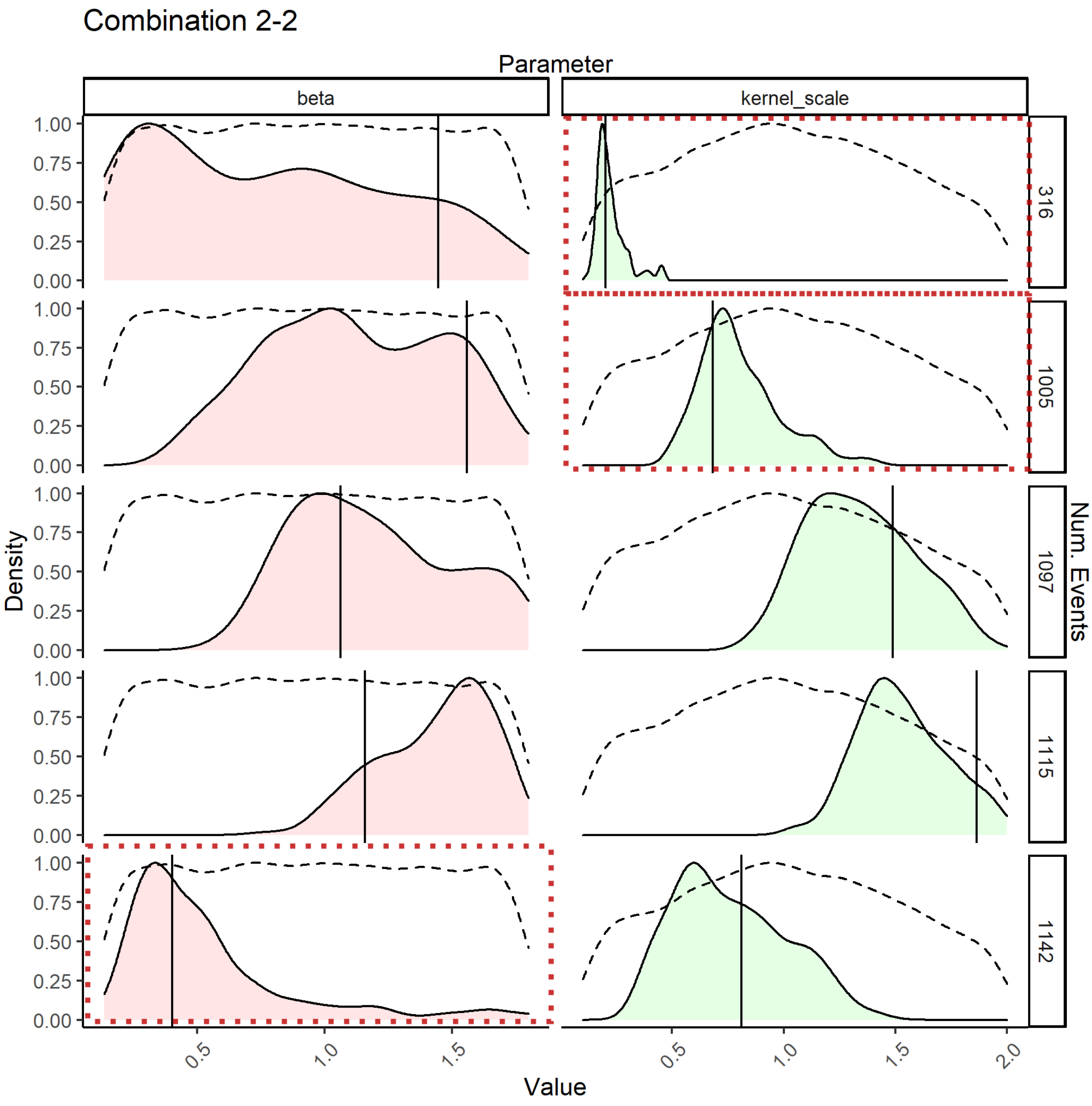

Figure S5: The density distributions of the parameters β and ν (**ID4-2-2**) after 6 generations of ABC-SMC. Each column is a different parameter, with colour-coded density plots. Each row corresponds to a different "true" value sampled from the prior distribution and recovery attempted via ABC-SMC. The black dotted line on each plot indicates the prior distribution for that parameter, and the vertical solid black line indicates the "true" sampled value for that particular parameter and fit. On the right-hand axis, the number indicate how many events were seen in that "true" outbreak, calculated as the total incidence of infection over the simulation. Higher totals indicate more information to fit to. Attempts where a parameter was considered recovered are surrounded by a dotted red box. With this set of parameters, β was recovered 1 out of 5 times, and ν was recovered 2 out of 5 times.

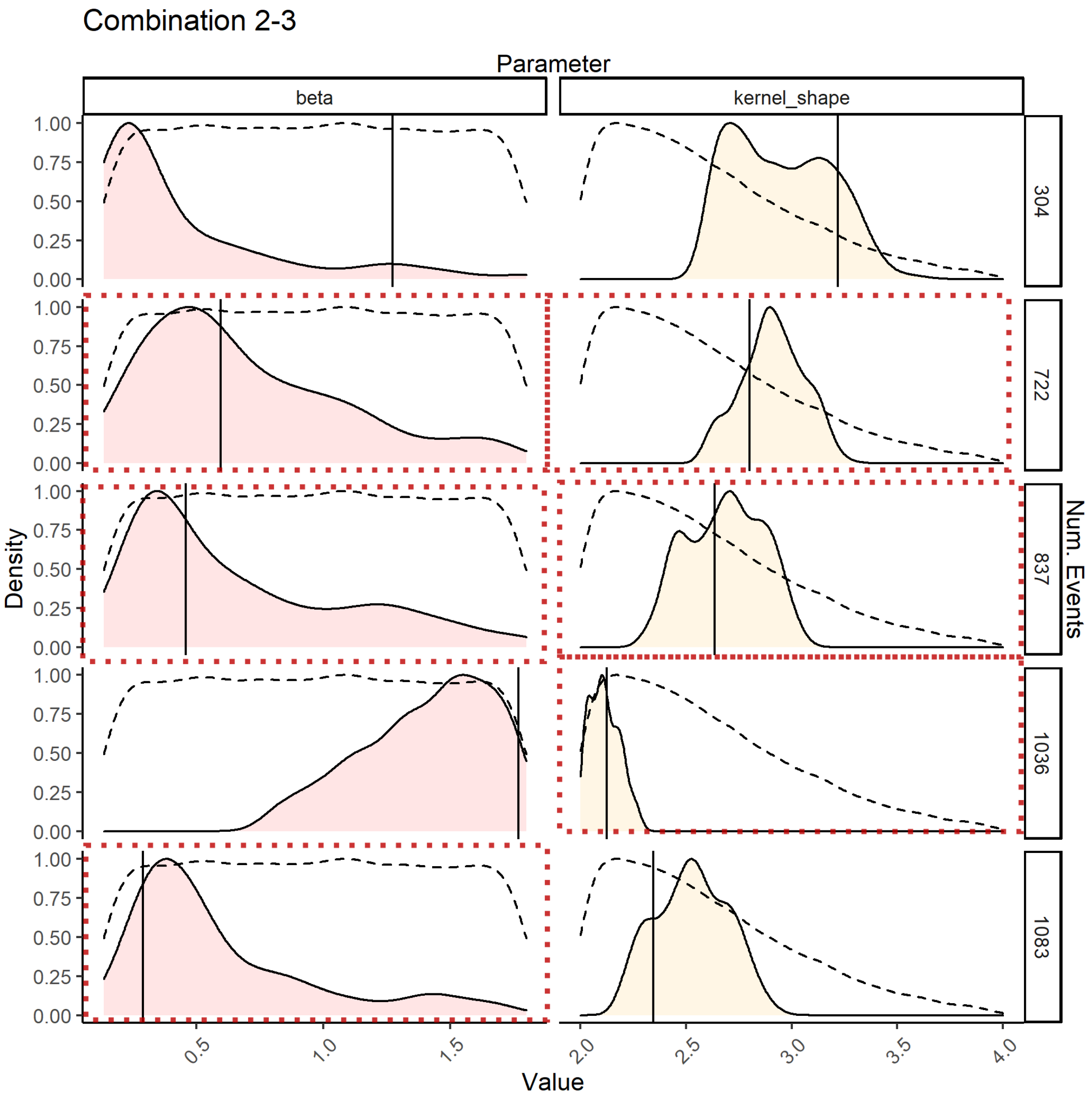

Figure S6: The density distributions of the parameters β and η (**ID4-2-3**) after 6 generations of ABC-SMC. Each column is a different parameter, with colour-coded density plots. Each row corresponds to a different "true" value sampled from the prior distribution and recovery attempted via ABC-SMC. The black dotted line on each plot indicates the prior distribution for that parameter, and the vertical solid black line indicates the "true" sampled value for that particular parameter and fit. On the right-hand axis, the number indicate how many events were seen in that "true" outbreak, calculated as the total incidence of infection over the simulation. Higher totals indicate more information to fit to. Attempts where a parameter was considered recovered are surrounded by a dotted red box. With this set of parameters, β was recovered 3 out of 5 times, and η was recovered 3 out of 5 times.

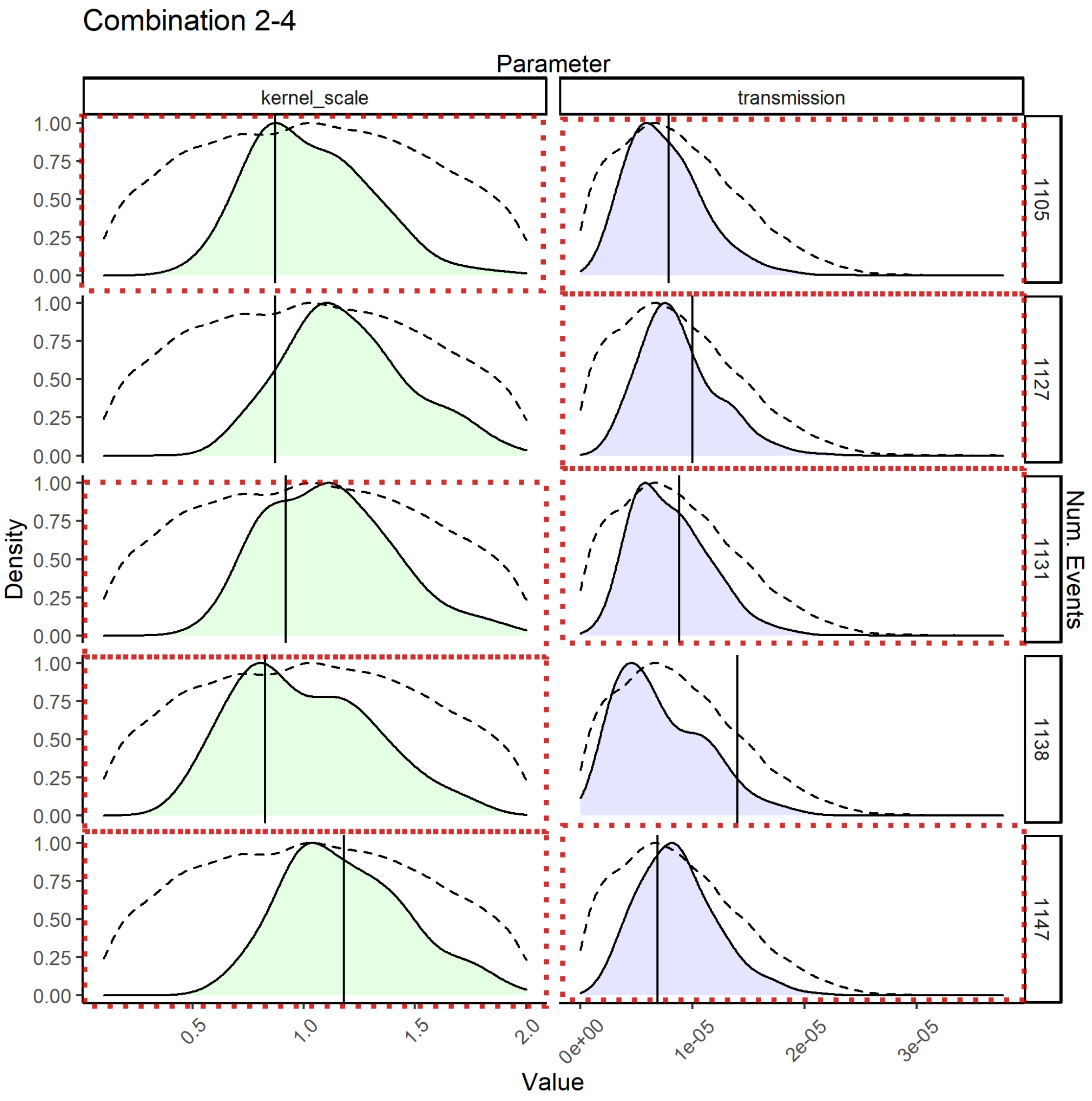

Figure S7: The density distributions of the parameters ν and ξ (**ID4-2-4**) after 6 generations of ABC-SMC. Each column is a different parameter, with colour-coded density plots. Each row corresponds to a different "true" value sampled from the prior distribution and recovery attempted via ABC-SMC. The black dotted line on each plot indicates the prior distribution for that parameter, and the vertical solid black line indicates the "true" sampled value for that particular parameter and fit. On the right-hand axis, the number indicate how many events were seen in that "true" outbreak, calculated as the total incidence of infection over the simulation. Higher totals indicate more information to fit to. Attempts where a parameter was considered recovered are surrounded by a dotted red box. With this set of parameters, ν was recovered 4 out of 5 times, and ξ was recovered 4 out of 5 times.

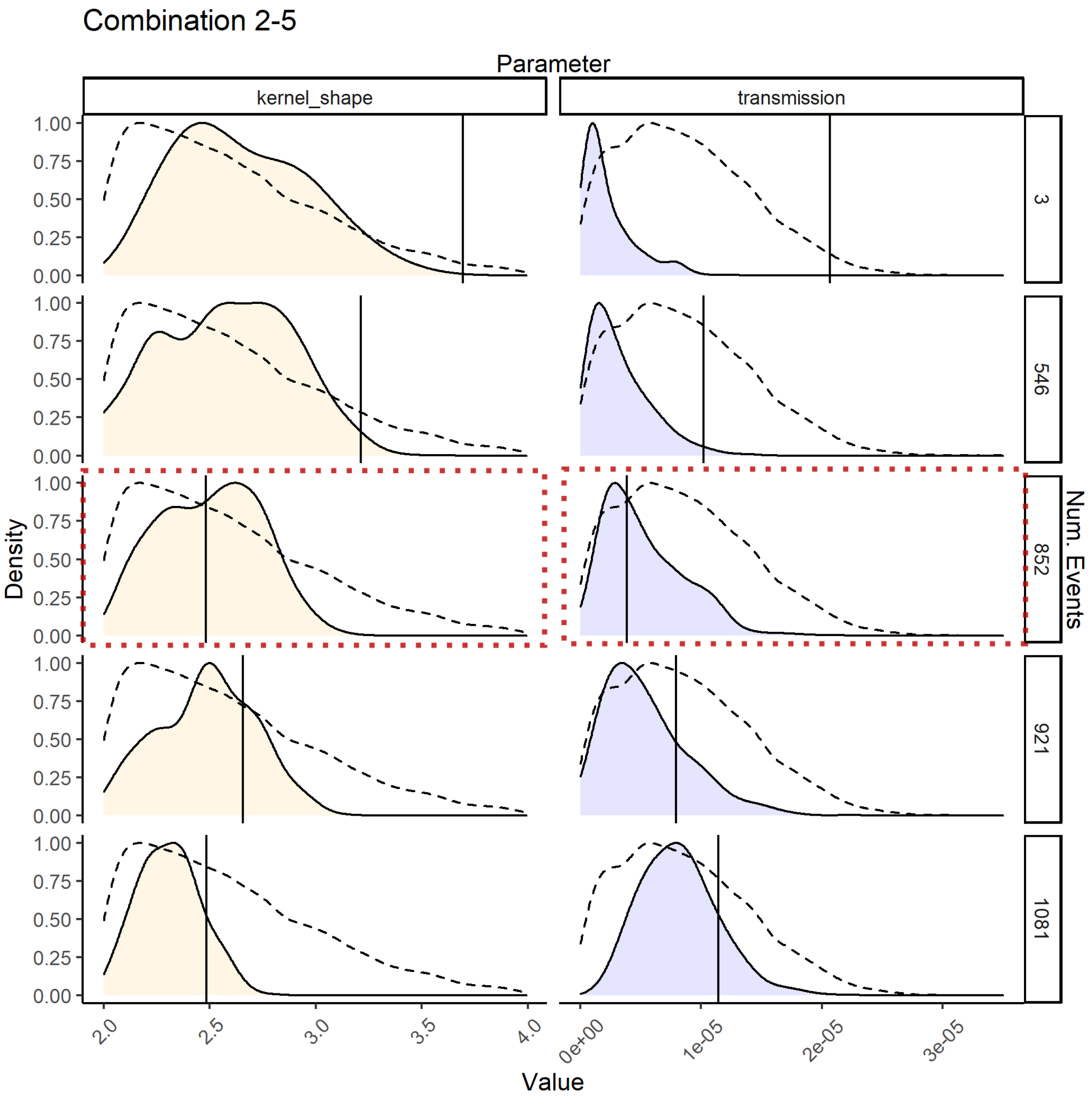

Figure S8: The density distributions of the parameters η and ξ (**ID4-2-5**) after 6 generations of ABC-SMC. Each column is a different parameter, with colour-coded density plots. Each row corresponds to a different "true" value sampled from the prior distribution and recovery attempted via ABC-SMC. The black dotted line on each plot indicates the prior distribution for that parameter, and the vertical solid black line indicates the "true" sampled value for that particular parameter and fit. On the right-hand axis, the number indicate how many events were seen in that "true" outbreak, calculated as the total incidence of infection over the simulation. Higher totals indicate more information to fit to. Attempts where a parameter was considered recovered are surrounded by a dotted red box. With this set of parameters, η was recovered 1 out of 5 times, and ξ was recovered 1 out of 5 times.

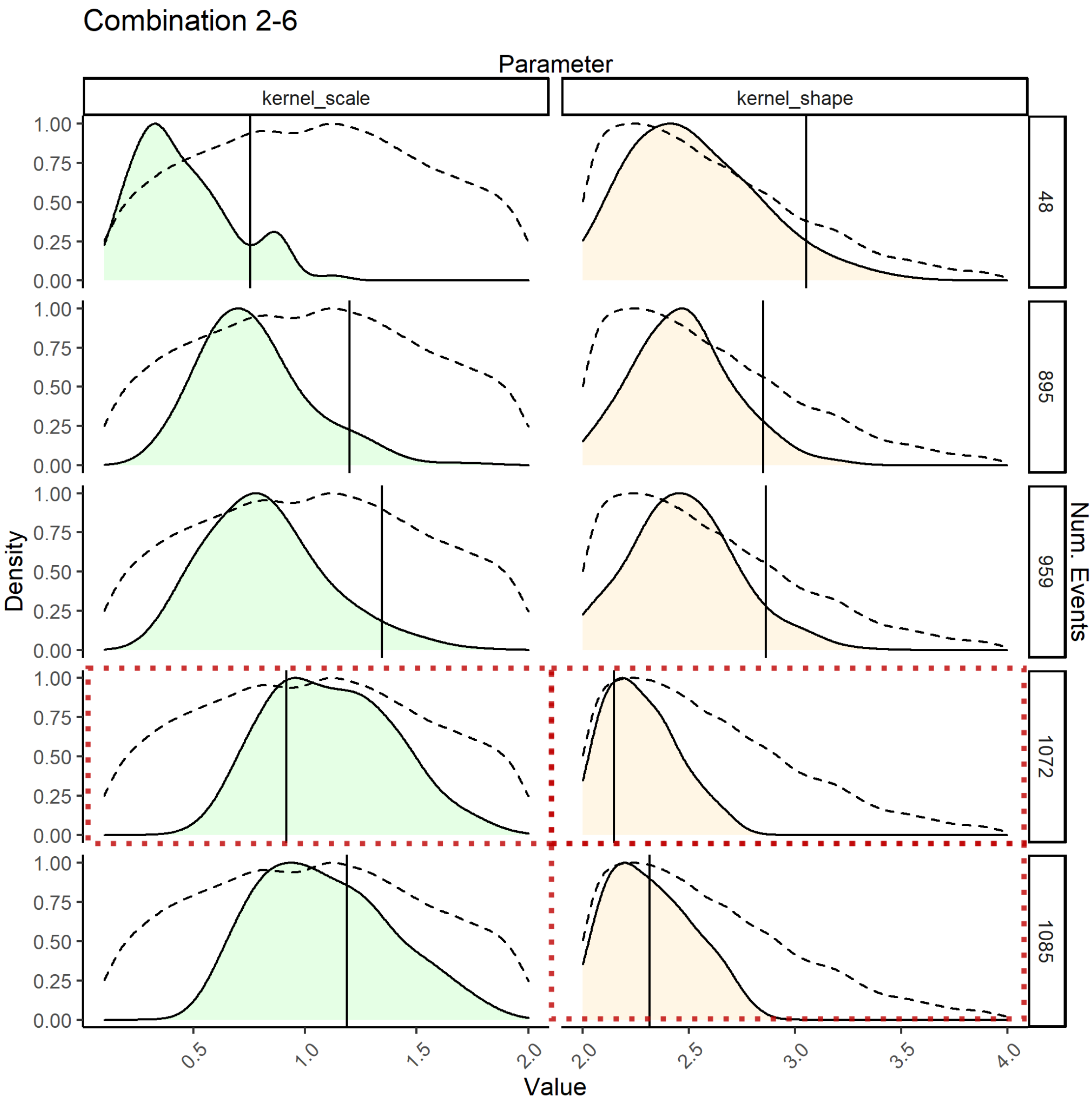

Figure S9: The density distributions of the parameters ν and η (**ID4-2-6**) after 6 generations of ABC-SMC. Each column is a different parameter, with colour-coded density plots. Each row corresponds to a different "true" value sampled from the prior distribution and recovery attempted via ABC-SMC. The black dotted line on each plot indicates the prior distribution for that parameter, and the vertical solid black line indicates the "true" sampled value for that particular parameter and fit. On the right-hand axis, the number indicate how many events were seen in that "true" outbreak, calculated as the total incidence of infection over the simulation. Higher totals indicate more information to fit to. Attempts where a parameter was considered recovered are surrounded by a dotted red box. With this set of parameters, ν was recovered 1 out of 5 times, and η was recovered 2 out of 5 times.

recovered once out of the 5 attempts.

Much better performance was seen with set **ID4-3-3** (fig. S12). This assessed ν, η, and ξ together, and the least identifiable parameter with this set of parameters was η, which was only recovered 3 out of the 5 attempts. Although no parameter was recovered every time, both ν and ξ were recovered 4 out of the 5 attempts.

Finally, set **ID4-3-4** (fig. S13) demonstrated decent performance, with β, η, and ξ all being recovered on 3 out of the 5 attempts.

The parameter set that demonstrated the greatest identifiability with the greatest number of parameters was parameter set **ID4-3-3**, which assessed kernel ν, η, and ξ together. It was this set of parameters that were used for the parameter estimation using the real data.

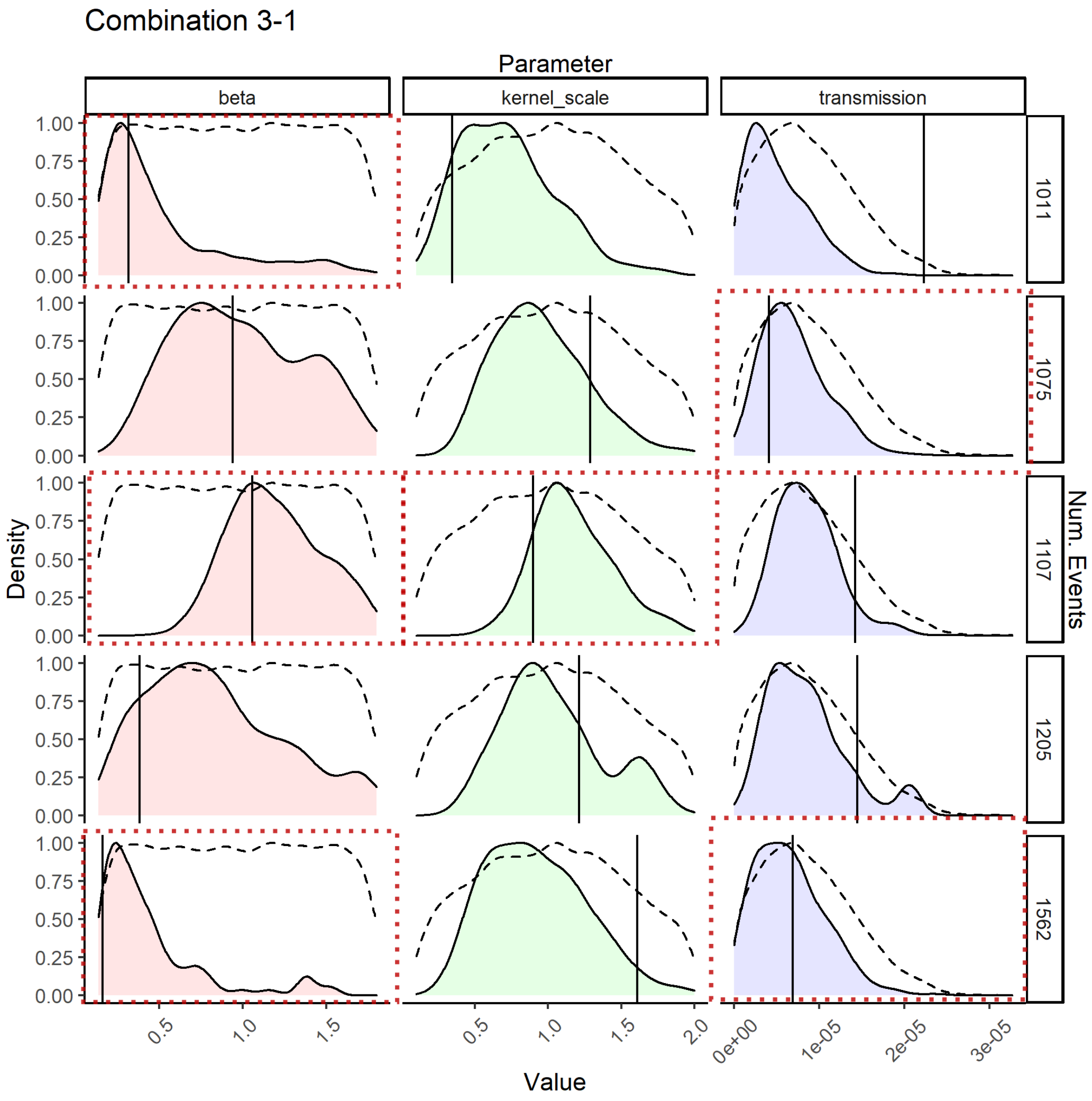

Figure S10: The density distributions of the parameters β, ν, and ξ (**ID4-3-1**), after 6 generations of ABC-SMC. Each column is a different parameter, with colour-coded density plots. Each row corresponds to a different "true" value sampled from the prior distribution and recovery attempted via ABC-SMC. The black dotted line on each plot indicates the prior distribution for that parameter, and the vertical solid black line indicates the "true" sampled value for that particular parameter and fit. On the right-hand axis, the number indicate how many events were seen in that "true" outbreak, calculated as the total incidence of infection over the simulation. Higher totals indicate more information to fit to. Attempts where a parameter was considered recovered are surrounded by a dotted red box. With this set of parameters, β was recovered 3 out of 5 times, ν was recovered 1 out of 5 times, and ξ was recovered 2 out of 5 times.

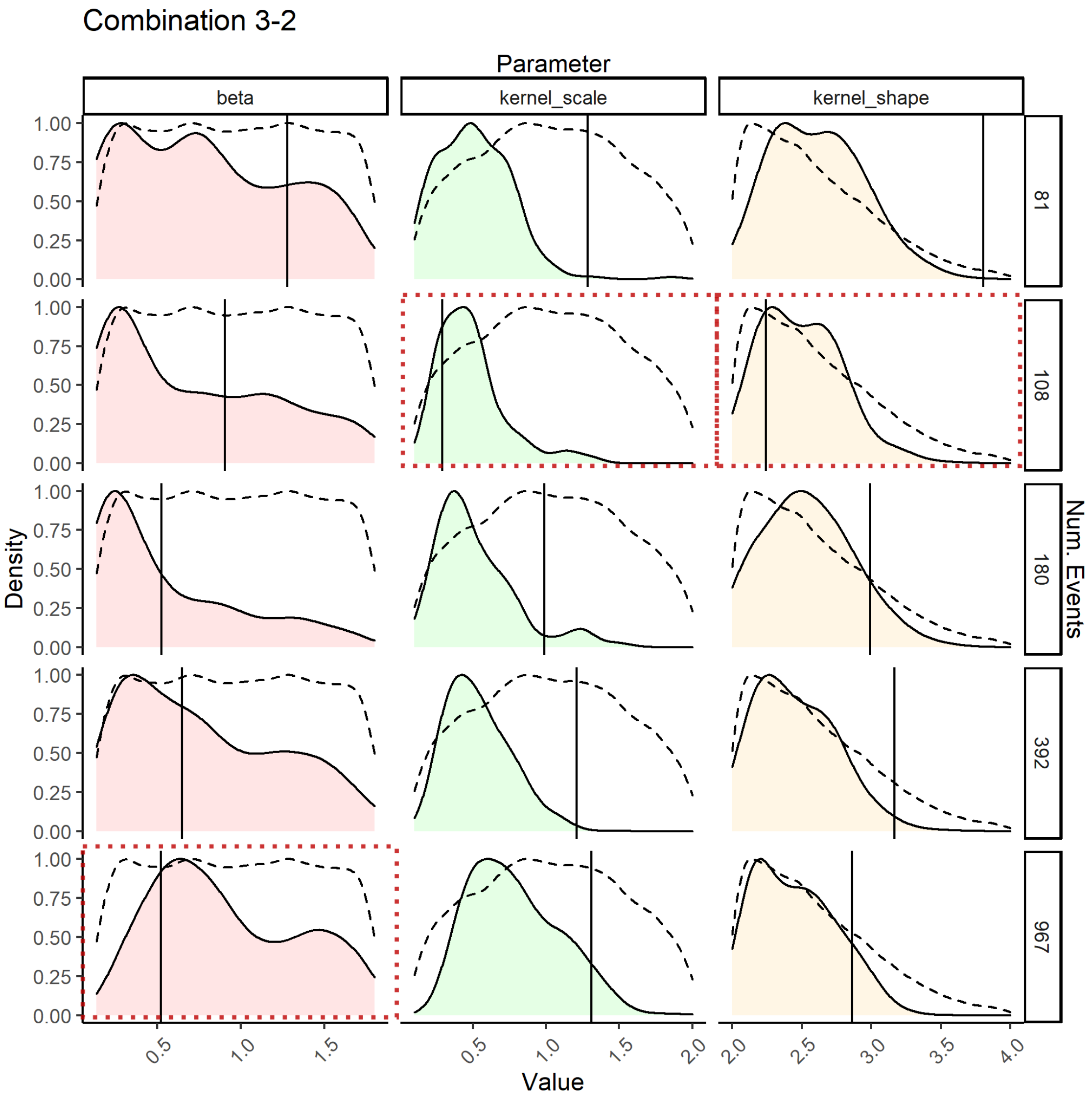

Figure S11: The density distributions of the parameters β, ν, and η (**ID4-3-2**) after 6 generations of ABC-SMC. Each column is a different parameter, with colour-coded density plots. Each row corresponds to a different "true" value sampled from the prior distribution and recovery attempted via ABC-SMC. The black dotted line on each plot indicates the prior distribution for that parameter, and the vertical solid black line indicates the "true" sampled value for that particular parameter and fit. On the right-hand axis, the number indicate how many events were seen in that "true" outbreak, calculated as the total incidence of infection over the simulation. Higher totals indicate more information to fit to. Attempts where a parameter was considered recovered are surrounded by a dotted red box. With this set of parameters, β was recovered 1 out of 5 times, ν was recovered 1 out of 5 times, and η was recovered 1 out of 5 times.

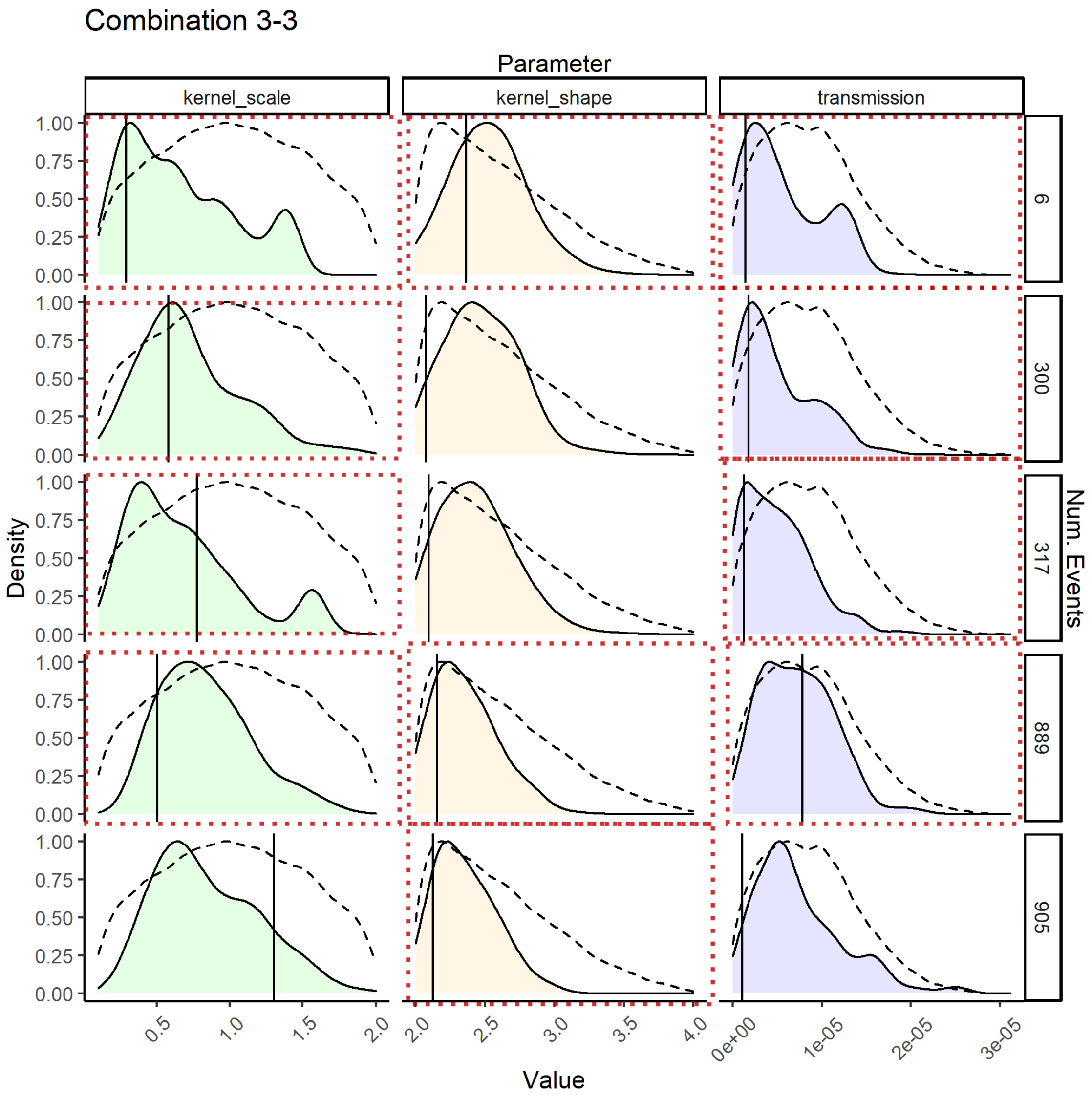

Figure S12: The density distributions of the parameters ν, η, and ξ (**ID4-3-3**) after 6 generations of ABC-SMC. Each column is a different parameter, with colour-coded density plots. Each row corresponds to a different "true" value sampled from the prior distribution and recovery attempted via ABC-SMC. The black dotted line on each plot indicates the prior distribution for that parameter, and the vertical solid black line indicates the "true" sampled value for that particular parameter and fit. On the right-hand axis, the number indicate how many events were seen in that "true" outbreak, calculated as the total incidence of infection over the simulation. Higher totals indicate more information to fit to. Attempts where a parameter was considered recovered are surrounded by a dotted red box. With this set of parameters, ν was recovered 4 out of 5 times, η was recovered 3 out of 5 times, and ξ was recovered 4 out of 5 times.

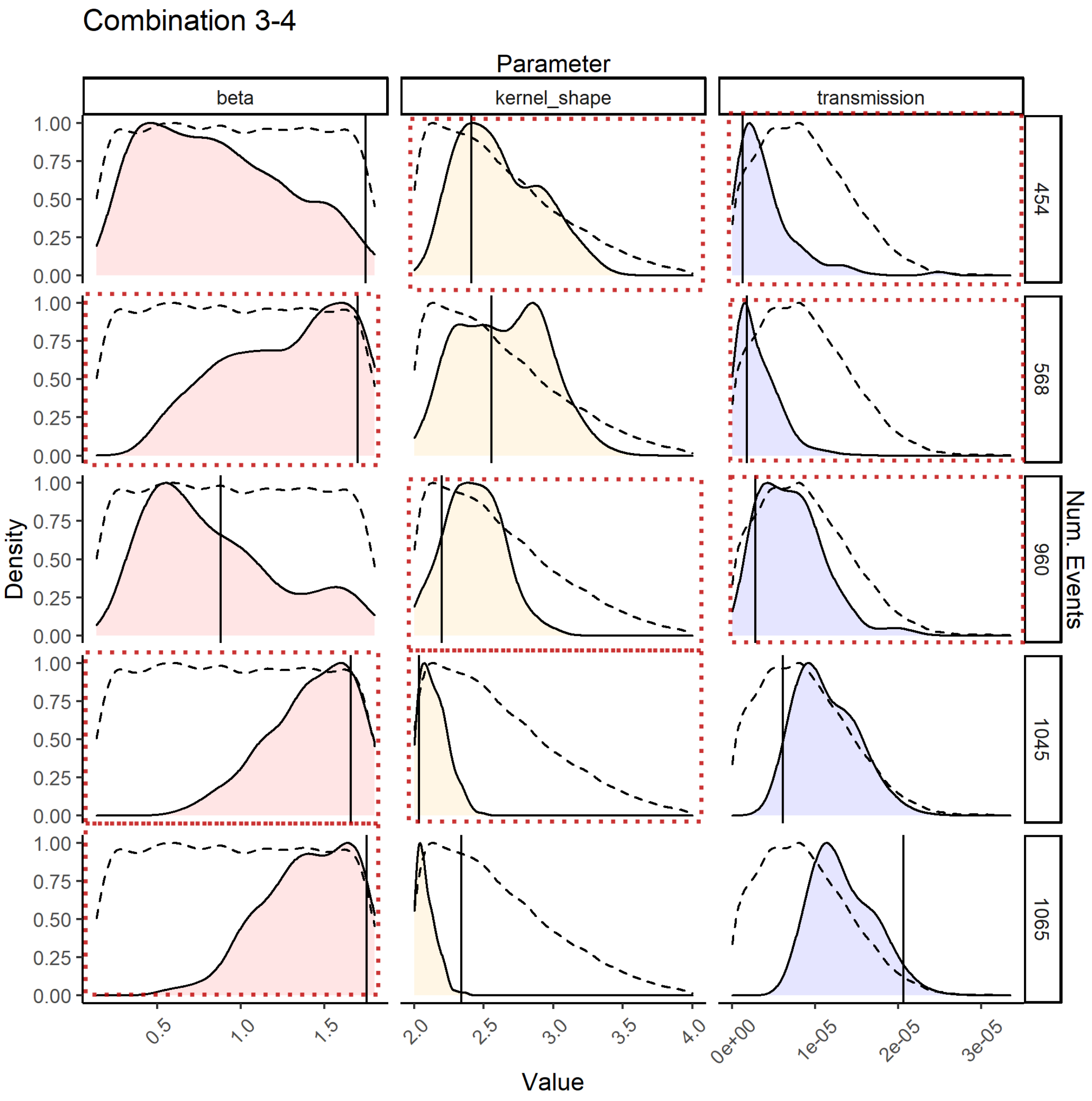

Figure S13: The density distributions of the parameters β, η, and ξ (**ID4-3-4**) after 6 generations of ABC-SMC. Each column is a different parameter, with colour-coded density plots. Each row corresponds to a different "true" value sampled from the prior distribution and recovery attempted via ABC-SMC. The black dotted line on each plot indicates the prior distribution for that parameter, and the vertical solid black line indicates the "true" sampled value for that particular parameter and fit. On the right-hand axis, the number indicate how many events were seen in that "true" outbreak, calculated as the total incidence of infection over the simulation. Higher totals indicate more information to fit to. Attempts where a parameter was considered recovered are surrounded by a dotted red box. With this set of parameters, β was recovered 3 out of 5 times, η was recovered 3 out of 5 times, and ξ was recovered 3 out of 5 times.
